# Mutational signatures of redox stress in yeast single-strand DNA and of aging in human mitochondrial DNA share a common feature

**DOI:** 10.1371/journal.pbio.3000263

**Published:** 2019-05-08

**Authors:** Natalya P. Degtyareva, Natalie Saini, Joan F. Sterling, Victoria C. Placentra, Leszek J. Klimczak, Dmitry A. Gordenin, Paul W. Doetsch

**Affiliations:** 1 Mutagenesis and DNA Repair Regulation Group, Laboratory of Genome Integrity and Structural Biology, National Institute of Environmental Health Sciences, National Institutes of Health, Durham, North Carolina, United States of America; 2 Mechanisms of Genome Dynamics Group, Laboratory of Genome Integrity and Structural Biology, National Institute of Environmental Health Sciences, National Institutes of Health, Durham, North Carolina, United States of America; 3 Integrative Bioinformatics Support Group, National Institute of Environmental Health Sciences, National Institutes of Health, Durham, North Carolina, United States of America; The University of Texas at Austin, UNITED STATES

## Abstract

Redox stress is a major hallmark of cancer. Analysis of thousands of sequenced cancer exomes and whole genomes revealed distinct mutational signatures that can be attributed to specific sources of DNA lesions. Clustered mutations discovered in several cancer genomes were linked to single-strand DNA (ssDNA) intermediates in various processes of DNA metabolism. Previously, only one clustered mutational signature had been clearly associated with a subclass of ssDNA-specific apolipoprotein B mRNA editing enzyme, catalytic polypeptide-like (APOBEC) cytidine deaminases. Others remain to be elucidated. We report here deciphering of the mutational spectra and mutational signature of redox stress in ssDNA of budding yeast and the signature of aging in human mitochondrial DNA. We found that the predominance of C to T substitutions is a common feature of both signatures. Measurements of the frequencies of hydrogen peroxide–induced mutations in proofreading-defective yeast mutants supported the conclusion that hydrogen peroxide–induced mutagenesis is not the result of increased DNA polymerase misincorporation errors but rather is caused by direct damage to DNA. Proteins involved in modulation of chromatin status play a significant role in prevention of redox stress–induced mutagenesis, possibly by facilitating protection through modification of chromatin structure. These findings provide an opportunity for the search and identification of the mutational signature of redox stress in cancers and in other pathological conditions and could potentially be used for informing therapeutic decisions. In addition, the discovery of such signatures that may be present in related organisms should also advance our understanding of evolution.

## Abstract 2

This study identifies mutational signatures of redox stress in yeast single strand DNA and in aged human mitochondrial DNA; analysis of data from The Cancer Genome Analysis project reveals the presence of redox stress-related mutational signatures in many tumors.

## Introduction

Oxidative stress is implicated in the etiology and progression of cancer via numerous and diverse mechanisms and pathways. Multiple studies have been aimed at gaining a comprehensive understanding of how dysregulation of cellular redox balance contributes to tumorigenesis and metastasis. Currently, the list of specific features of redox disbalance important for carcinogenesis is far from complete. It is now well established that inflammation caused by prolonged activation of reactive oxygen species (ROS)-producing immune cells in response to chronic viral and bacterial infection [1, 2, 3] contributes to the development of cervical, liver, gastric, and other cancers. The mechanisms of tumor initiation by chronic infection are very complex and are believed to be an indirect consequence of deregulation of multiple pathways in host tissues by infectious agents as well as an aberrant, prolonged activation of the immune system. Only recently has direct evidence for the causative role of excessive production of ROS by myeloid cells in transformation of neighboring epithelial cells been demonstrated in a mouse intestinal tumor model [4]. In the same study, the contribution of ROS to metastasis was assessed, and it was concluded that increased levels of ROS promote tumor progression and invasion. However, a number of seemingly contradictory reports argue that under different circumstances, ROS can prevent cancer progression by stimulation of apoptosis [5], inhibition of metastasis [6], and potentiation of tumoricidal capability of immune cells [7]. Tumor cells, compared to normal cells, are burdened with higher levels of ROS and thus rely on up-regulation of antioxidant pathways for survival, which makes them vulnerable to antioxidant inhibition. Accordingly, down-regulation of antioxidant systems impairs initiation of mammary tumors and decreases the tumor burden in mouse models of spontaneous sarcomas and lymphomas [8]. Considering the complexity and intricate cross-talk between systems that maintain redox status in cancer cells and their microenvironment, it is not surprising that experimental and epidemiological data on the efficacy of either administration of antioxidants or, vice versa, inhibition of antioxidants in cancer treatment/prevention is very heterogeneous. In general, there is no clear evidence that antioxidants could prevent or reduce cancer development and progression [9]. At the same time, clinical trials involving targeted inhibition of antioxidant glutathione did not provide any support for the potential utility of increasing ROS levels for anticancer treatment [10]. While current research indicates that oxidative stress can have either a positive or negative effect on individual cancer prognosis, there is a growing expectation that a more detailed understanding of the consequences of redox stress in different types of tumors may inform specific therapeutic interventions and dietary recommendations in personalized cancer treatments. However, this approach would require an estimation of the redox stress levels in a tumor of an individual. For instance, if sequencing of a patient’s cancer genome and analysis of the mutational signatures reveals extensive oxidative damage, a therapy directed at inhibition of antioxidant systems might be productive and should be considered, whereas in cases in which the signature does not display mutational signature of oxidative DNA damage, antioxidants could be beneficial in preventing tumor progression.

Currently, 40 mutation signatures for cancers have been identified, and the underlying molecular mechanisms for some signatures have been elucidated (http://cancer.sanger.ac.uk/cosmic, [11]). On the other hand, recent experiments confirmed that exposure of cultured mouse cells to two different mutagenic agents, ultraviolet light and benzo[a]pyrene, resulted in genome-wide mutational signatures attributable to these agents [12]. Even though the analysis of the majority of cancer signatures does not provide an immediate understanding of the underlying mechanisms for tumorigenesis, discovery of single-strand DNA (ssDNA)-specific apolipoprotein B mRNA editing enzyme, catalytic polypeptide-like (APOBEC3)-induced mutation signatures indicated that the persistent presence of ssDNA is an important, distinguishing feature of cancer genomes [13]. In addition, changes in DNA transactions in transformed cells such as replication fork uncoupling [14], deregulation of deoxynucleotide triphosphate (dNTP) pools [15], and replicative stress in general, could increase the ssDNA burden of the cancer genomes compared to that of nontransformed cells. Moreover, ssDNA, unlike double-stranded DNA (dsDNA), is prone to accumulating multiple, closely spaced mutations occurring with high frequency [16, 17]. This property of ssDNA may facilitate deciphering mutational signatures induced by a specific mutagen in experimental settings. Thus, information about the mutational signature of redox stress–induced oxidative damage in ssDNA can be useful for defining the sources of mutation burden in cancer genomes.

Identifying mutational signatures caused by redox stress in ssDNA may also shed light on the age-dependent accumulation of mutations in mitochondrial DNA (mtDNA). For more than 60 years, oxidative damage has been inferred to be a cause of aging [18]. However, accumulating experimental evidence suggests that oxidative stress does not always accelerate aging and that expression of antioxidants in some systems decreases lifespan and leads to deleterious consequences (reviewed in [19, 20]). The hypothesis that the burden of oxidative damage increases with age in mtDNA was recently refuted based on the absence of an increase in the frequency of G to T transversions in mitochondrial DNA of aged humans and flies [21, 22]. Despite the ongoing debate on the precise mechanism of mitochondrial replication [23, 24], biochemical studies indicated that the synthesis of leading and lagging strand DNA in mitochondria becomes uncoupled, at least temporarily [25], and even though stretches of ssDNA can be protected by RNA and/or mitochondrial ssDNA–binding protein, such uncoupling increases the probability of exposure to ROS produced by mitochondrial metabolism. If this is the case, one might expect that the mutational signature of aging in mtDNA may be similar to the signature of oxidative DNA damage in ssDNA.

We have previously shown that oxidative damage can cause mutagenesis in a yeast subtelomeric ssDNA reporter and was detectable despite a high background of spontaneous mutagenesis in ssDNA [26]. However, this reporter system was not sensitive enough to allow for identification of the distinct mutational signature of oxidative damage in ssDNA. In this study, we employed a more sensitive reporter system in *Saccharomyces cerevisiae*, allowing direct selection for mutation clusters in ssDNA. This approach enabled identification of the cellular systems involved in prevention of redox stress–induced mutagenesis in ssDNA and facilitated the search for mutational signatures.

We report here the elucidation of the mutational signature of oxidative stress in yeast ssDNA as well as a mutational signature of aging in human mtDNA, which shares similarity with the yeast signature, with respect to the major type of base substitutions. These findings provide insight into the contribution of redox stress in cancer initiation, progression, and metastasis, as well as in the process of aging.

## Results

### Genetic system for deciphering the mutational signature of oxidative DNA damage

**Fig 1:**
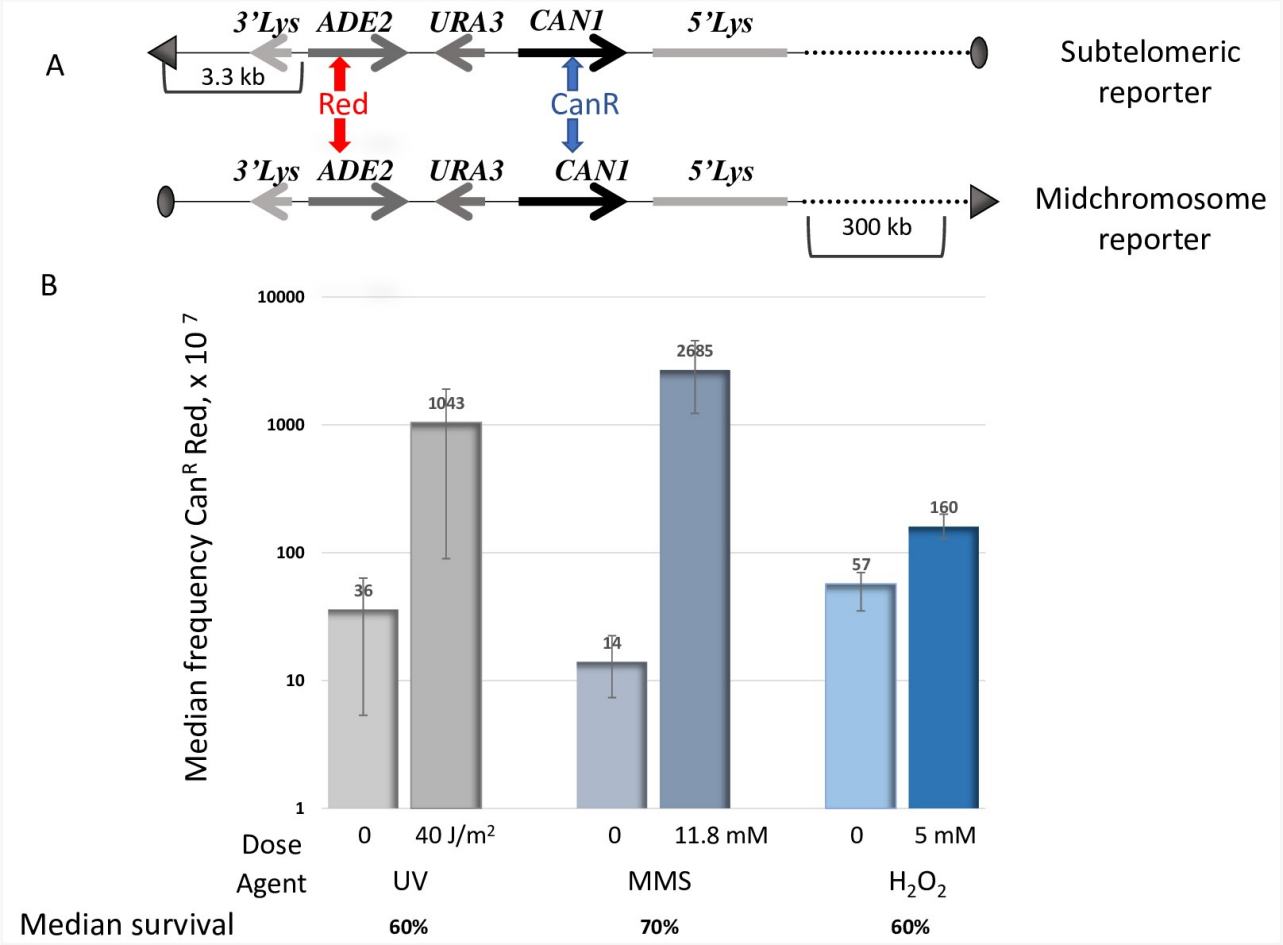
Exogenous DNA-damaging agents induce closely spaced mutations in ssDNA. A. Schematic representation of the triple-reporter system for direct detection of clustered mutations in ssDNA. Subtelomeric reporter: *CAN1*, *URA3*, and *ADE2* genes were placed within disrupted *LYS2* gene, close to de novo constructed telomere at Chromosome V in the *cdc13-1ts* mutant strain. At 37°C, uncapping of telomeres and prevention of resynthesis of the resected telomeres in *cdc13-1ts* cells results in persistent ssDNA comprising the reporter genes up to 6 hours without significant loss of viability. Simultaneous mutations in *CAN1* (blue vertical arrows) and *ADE2* (red vertical arrows) give rise to CanR Red colonies. Midchromosome reporter: *CAN1*, *URA3*, and *ADE2* genes were placed in the middle of the right arm of Chromosome II, more than 300 kb away from either telomere. The horizontal arrowheads show the direction of the transcription of the corresponding genes. Telomeres are depicted as triangles and centromeres as ovals. B. In ssDNA, oxidative DNA-damaging agent, hydrogen peroxide, at a dose equitoxic to that of MMS and UV, induces less closely spaced (CanR Red) mutations than either of these mutagens. All experiments were carried out as described in Materials and methods. Mutation frequencies for six to 12 independent cultures were measured in each experiment. Median frequencies of spontaneous mutations for each type of exposure are shown separately because the technical settings of these experiments were slightly different: in order to achieve the same level of toxicity for different mutagens, the time of the exposure to MMS and mock exposure in the corresponding controls was adjusted to 30 minutes, whereas the time of incubation of the cells with hydrogen peroxide and the corresponding mock incubation in water lasted 2 hours. Therefore, the longer time of persistence of ssDNA in the controls for hydrogen peroxide exposure could have contributed to an elevated basal level of spontaneous mutations, compared to the levels of mutations spontaneous in experiments involving MMS. Whiskers represent 95% confidence limits for median frequency of mutations. See also S1 Data. CanR Red, canavanine-resistant red; H_2_O_2_, hydrogen peroxide; MMS, methyl methanesulfonate; ssDNA, single-strand DNA; UV, ultraviolet irradiation.

To decipher the signature of redox stress–induced oxidative DNA damage in ssDNA, we utilized a yeast reporter system optimized for detecting clustered mutation events that result in inactivation of closely spaced *CAN1* and *ADE2* genes (see Fig 1 in [27] and Fig 1A). The triple-reporter system is comprised of intact *CAN1*, *URA3*, and *ADE2* genes, inserted into the *LYS2* locus. Incubation of the *cdc13-1* temperature-sensitive mutants at 37°C causes telomere uncapping followed by 5′→3′ resection at the ends of chromosomes, encompassing several kilobases of subtelomeric regions, thus allowing transient formation of long ssDNA stretches at the nonpermissive temperature. The *CAN1/URA3/ADE2* triple reporter was placed in one of two locations in the genome of the *cdc13-1* strains. For assessing mutagenesis in ssDNA, the reporter was inserted at a subtelomeric location, where it is frequently encompassed by resection. For assessment of mutagenesis in dsDNA, the triple reporter was placed at a midchromosomal location, greater than 300 kb away from the right telomere on Chromosome II, where the reporter remains double stranded even when resection is stimulated by a shift to the nonpermissive temperature [27]. Canavanine-resistant red colonies (CanR Red) can arise as a consequence of at least two mutations, one in *CAN1*, allowing growth on the media containing canavanine, and another in *ADE2*, making the colonies red or pink, thereby visually distinguishable from white, Ade^+^, colonies. Sanger sequencing of these two genes as well as the adjacent *URA3* and *LYS2* sequences of CanR Red isolates in strains with the subtelomeric reporter enables analysis of the mutation spectra, density, and distribution patterns of mutations caused by endogenous and exogenous DNA-damaging agents in ssDNA.

Utilizing the subtelomeric triple reporter, we measured the frequencies of mutation clusters caused by exposure to two potent ssDNA mutagens, ultraviolet (UV) irradiation, and methyl methanesulfonate (MMS) [28] and compared them to frequencies of hydrogen peroxide–induced mutation clusters in ssDNA. Following exposure to moderately toxic doses (60%–70% survival) of these DNA-damaging agents, the increase in the mutation frequency induced by hydrogen peroxide was quite modest compared to increases induced by MMS and UV irradiation at equitoxic doses (Fig 1B, S1 Data). These data confirm our previous observation [26] and indicate that ssDNA in yeast cells is well protected from oxidative damage. It had been shown that deletion of the major reactive oxygen scavenging enzymes, catalase and superoxide dismutase, do not increase spontaneous mutation rates in ssDNA. Moreover, exposure to exogenous oxidative agents increased the mutation frequencies in ssDNA only slightly [26]. Such low increases presented an apparent obstacle in the search for a mutational signature because identification of such a signature requires analysis of numerous independent mutants; the larger the number of the mutants analyzed, the higher the confidence in the putative signature. Therefore, we searched for the genes protecting ssDNA from mutagenesis by oxidative damage.

### Cellular regulators of ssDNA mutagenesis

We sought to identify genes involved in preventing redox stress–induced instability in ssDNA in order to i) uncover the mechanisms for protection of a vulnerable target such as ssDNA from redox stress–induced oxidative damage and ii) to find genetic backgrounds allowing for selection of high numbers of the oxidative stress–induced mutations. Assuming that the candidate genes will be involved in the maintenance of the cellular redox balance, we constructed a number of single, double, and triple mutants and screened them for increased sensitivity to hydrogen peroxide mutagenesis (S1 Table). None of the strains tested displayed an increase in hydrogen peroxide–induced mutations that was comparable to the increase in mutation frequencies caused by MMS or UV irradiation. However, unexpectedly, modulation of the cellular chromatin structure by exposure of the cells to nicotinamide (NAM), a nonspecific inhibitor of histone deacetylases [29], dramatically increased levels of spontaneous mutations in ssDNA, even though it did not affect hydrogen peroxide–induced mutagenesis (S1A Fig, S1 Data). This finding suggested that chromatin status can change the accessibility of ssDNA to endogenous sources of DNA damage. Accordingly, we explored the potential involvement of genes regulating nuclear DNA packaging in ssDNA mutagenesis. Histone deacetylases Hst3 and Hst4 are important regulators of genome stability and play a significant role in prevention of spontaneous mutagenesis in dsDNA. Although deletion of either of these two genes did not alter the mutation frequencies, the double mutant showed a synergistic increase in frequencies [30]. As in the case with dsDNA, deletion of either of these two genes did not change the mutation frequencies at the ssDNA reporter, supporting the conclusion that their functions are redundant. Spontaneous mutation frequencies in double mutant *hst3Δ hst4Δ* were higher than in single mutants (S1B Fig, upper panel; S1 Data). However, in the *cdc13-1ts* mutant background at the nonpermissive temperature (37°C), survival of the double mutant decreased significantly (S1B Fig, lower panel; S1 Data), making it impossible to attribute the increase in mutation accumulation only to oxidative DNA damage.

**Fig 2:**
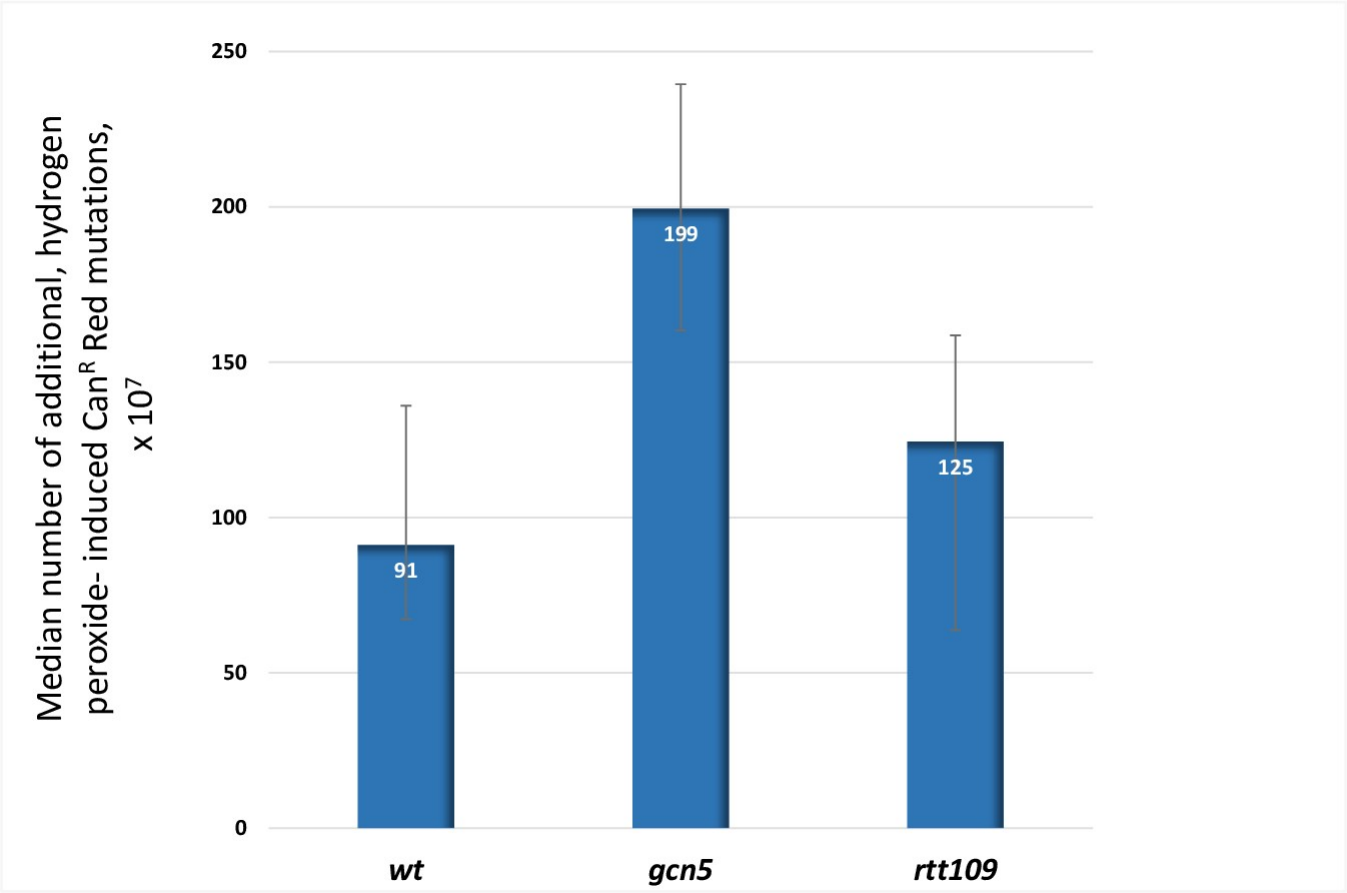
Exposure to hydrogen peroxide leads to significant increase in frequencies of additional (compared to spontaneous) closely spaced (CanR Red) mutations in ssDNA in the *gcn5* mutant. Mutation frequency measurement experiments were set up in such a way that each individual culture had been split and either exposed or mock exposed to hydrogen peroxide (Materials and methods). The number of additional hydrogen peroxide–induced mutations in each culture can be calculated by subtraction of the frequencies of spontaneous mutations from the frequencies of induced mutations. Utilization of the number of additional peroxide-induced mutations as a parameter for comparison allows for better control of the differences in technical repeats of the experiment when the slight differences in the conditions could make the base level of the mutations differ from one repetition to another. Whiskers represent 95% confidence limits for median of mutation frequencies. The median frequency of hydrogen peroxide–induced, closely spaced CanR Red mutants is significantly increased in *gcn5* compared to wild type (*P* = 0.0014 by Mann–Whitney test). See also S1 Data. CanR Red, canavanine-resistant red; ssDNA, single-strand DNA.

Acetyltransferase Gcn5 and its functional homolog Rtt109 play multiple roles in maintaining genomic stability, as well as in other processes of DNA metabolism [31, 32]. Both acetyltransferases were implicated in the deposition of histone H3–H4 onto unwound ssDNA behind the replication fork [33]. In dsDNA (midchromosome reporter), frequencies of hydrogen peroxide–induced mutations in *gcn5* are higher than in *wild-type* (*wt*) and *ogg1* strains (S2A Fig, S2 Table, S1 Data). In ssDNA, the median frequency of hydrogen peroxide–induced, closely spaced CanR Red mutants was significantly increased in *gcn5* compared to wild type (*P* = 0.0014) (Fig 2, S2B Fig, S1 Data). Even though the frequencies of additional mutations in *rtt109* strains were not higher than in *wt* strain, the density of nonselected mutations in *gcn5*, as well as in r*tt109* strains, was higher than in the *wt* strain: 1 per 9.3 kb and 1 per 10.1 kb in *rtt109* and in *gcn5* mutants, respectively, as compared to 1 per 14.5 kb in the *wt* strain (S3 Table). Furthermore, the majority of CanR Red isolates derived from the *wt* strains contained only two mutations (one in *CAN1* and one in *ADE2*). Whereas in *rtt109* and *gcn5* strains, the majority of CanR Red isolates harbored additional mutations (*P* = 0.04) (S3A Fig, S1 Data). The distribution of mutations within the triple reporter in *rtt109* and *gcn5* strains differs from that of the *wt* strain (S3B Fig, S1 Data). The distance between neighboring mutations in *rtt109* and *gcn5* strains is smaller than that in *wt* and *ogg1* strains (S3C Fig, S1 Data). Another unusual feature of the mutation distribution in strains with perturbed chromatin structure, in *rtt109* and *gcn5* strains, is an enrichment for mutations separated by less than 147 bp (approximate length of dsDNA wrapped around one nucleosome). Such closely spaced mutations were detected in only two of 50 hydrogen peroxide–induced CanR Red isolates derived from the *wt* strain (S4A Fig), while in *rtt109* (S4B Fig) and *gcn5* mutants (S4C Fig), the corresponding numbers were higher (*P* = 0.03, one-sided Fisher exact test): six of 47 and eight of 50, respectively. Taken together, these data demonstrate that vulnerability of ssDNA to oxidative damage can be modulated by the Rtt109 and Gcn5 histone acetylases. Utilization of *gcn5*- and *rtt109*-derived CanR Red mutants for analysis of mutational signature of oxidative DNA damage in ssDNA enhanced the detection of the closely spaced mutants and facilitated the analysis of the mutational signatures.

To rule out the possibility that in *gcn5* and *rtt109* mutants and increase in density and in frequencies of additional hydrogen peroxide–induced mutations is caused by expansion of the length and/or by longer persistence of subtelomeric ssDNA, as opposed to facilitation of redox stress–induced mutagenesis, we measured induction of CanR Red mutations by a different type of DNA-damaging agent: UV light. We observed that following UV exposure, *rtt109* mutants produced fewer clustered mutations (CanR Red) as compared to *wt* strains (S5 Fig, S1 Data). These data argue that the modulation of chromatin status by these histone acetylases leading to protection of DNA from redox stress–induced mutagenesis does not involve changes in dynamics of telomere resection.

### Hydrogen peroxide mutagenesis is not caused by an increased frequency of replicative DNA polymerase errors

**Fig 3:**
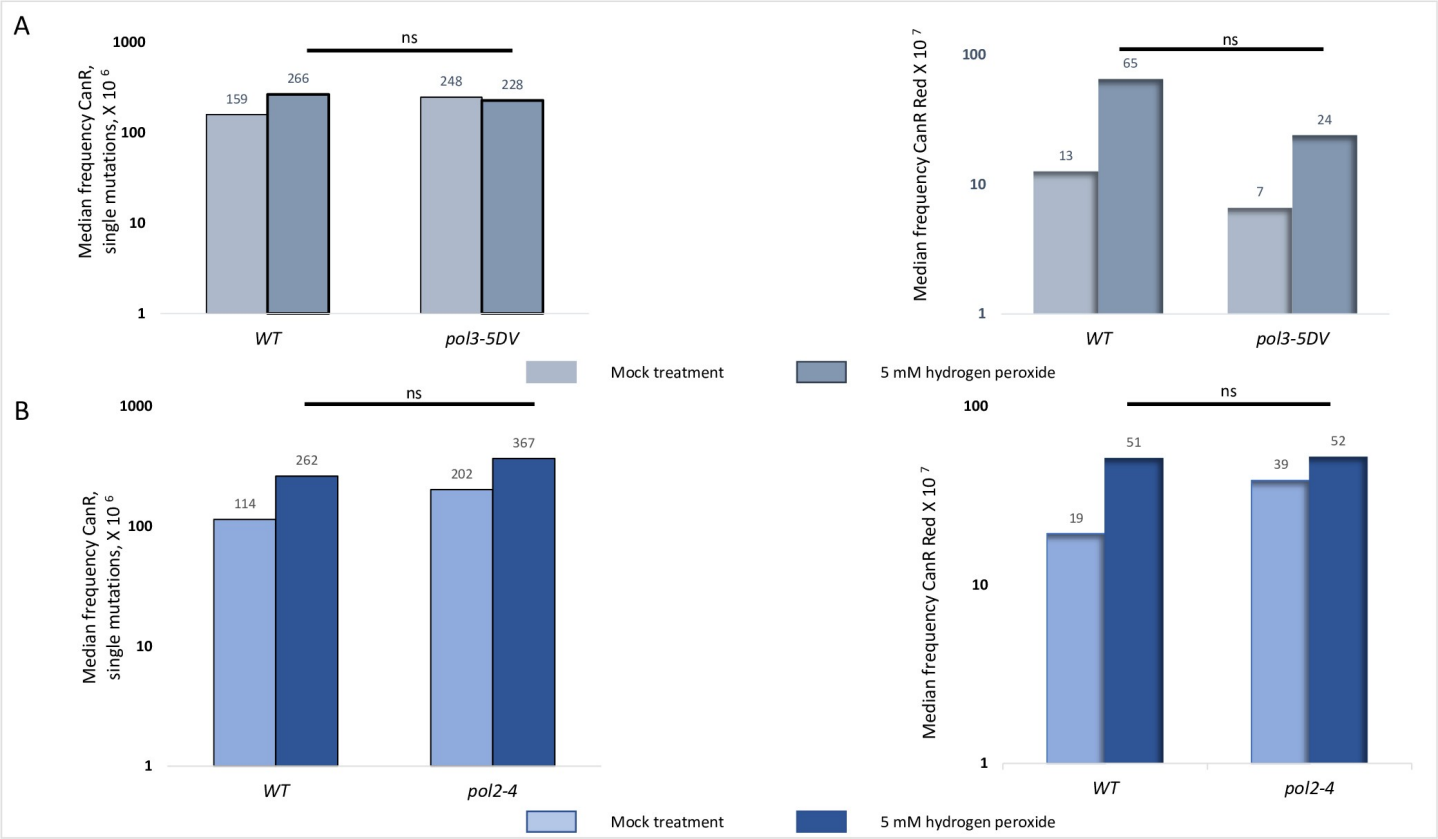
Hydrogen peroxide–induced mutagenesis is not caused by increased rates of incorporation errors by DNA polymerases delta and epsilon. A. Frequency of spontaneous and peroxide-induced CanR mutations and CanR Red closely spaced mutations in ssDNA triple reporter in parental wild-type and derivate *pol3-5DV* strains had been measured simultaneously for four (*wt*) and 24 (*pol3-5DV*) independent cultures, as described in Materials and methods. *P* values were determined by the Mann–Whitney test. See also S1 Data. B. Frequency of spontaneous and peroxide-induced CanR mutations and CanR Red closely spaced mutations in ssDNA triple reporter in parental wild-type and derivate *pol2-4* strains had been measured simultaneously for eight (*wt*) and eight (*pol2-4*) independent cultures, as described in Materials and methods. *P* values were determined by the Mann–Whitney test. See also S1 Data. CanR Red, canavanine-resistant red; ssDNA, single-strand DNA; wt, wild-type.

Hydrogen peroxide is highly reactive chemical, and it is possible that it exerts its mutagenic effects toward ssDNA indirectly by damaging a replicative DNA polymerase involved in resynthesis of the second strand in the long stretch of subtelomeric ssDNA. Eukaryotic DNA polymerases require iron sulfur clusters to form active replicating complexes [34]. These clusters are extremely ROS sensitive and some of the cellular proteins containing them are ROS labile [35]. If hydrogen peroxide is causing mutations by increasing replicative polymerase misincorporation rates, hydrogen peroxide exposure of the strains with a polymerase proofreading defect should increase mutagenesis in the subtelomeric ssDNA reporter, compared to strains with wild-type proofreading, exposed to hydrogen peroxide. In other words, if oxidatively damaged DNA polymerase misincorporates with high frequency and, in addition, cannot correct these mistakes due to lack of proofreading activity, then in proofreading-defective mutants, hydrogen peroxide will induce mutations at a higher frequency than in *wt* strains. To address this possibility, we measured ssDNA mutation frequencies in the strains with compromised proofreading activities of the major DNA polymerases: mutant DNA polymerase epsilon *pol2-4* [36] or mutant DNA polymerase delta *pol3-5DV* [37]. In all of the polymerase proofreading-defective strains tested, the frequencies of hydrogen peroxide–induced CanR single mutations in ssDNA, presented in the left panels of Fig 3A and Fig 3B, and CanR Red closely spaced mutations, presented in right panels of Fig 3A and Fig 3B, did not differ from the frequency of hydrogen peroxide–induced mutations in the *wt* strain. These data suggest that hydrogen peroxide causes mutagenesis by damaging ssDNA directly rather than by damaging the DNA polymerases and increasing DNA polymerases error rates. To rule out the possibility that defects in proofreading activity of DNA polymerase changes the proportion of gross chromosomal rearrangements (GCRs) (loss of the subtelomeric region between *CAN1* locus and telomere, which would also lead to the appearance of CanR Red mutants), we estimated the frequencies of non-GCR events by counting Ura^+^ colonies among all CanR Red colonies. The resulting estimated frequencies did not change the conclusion that the increase in hydrogen peroxide–induced clustered mutations cannot be explained by an increase in errors made by replicative DNA polymerases (S4 Table).

### Distinct signature of redox stress–induced mutagenesis in ssDNA

**Fig 4:**
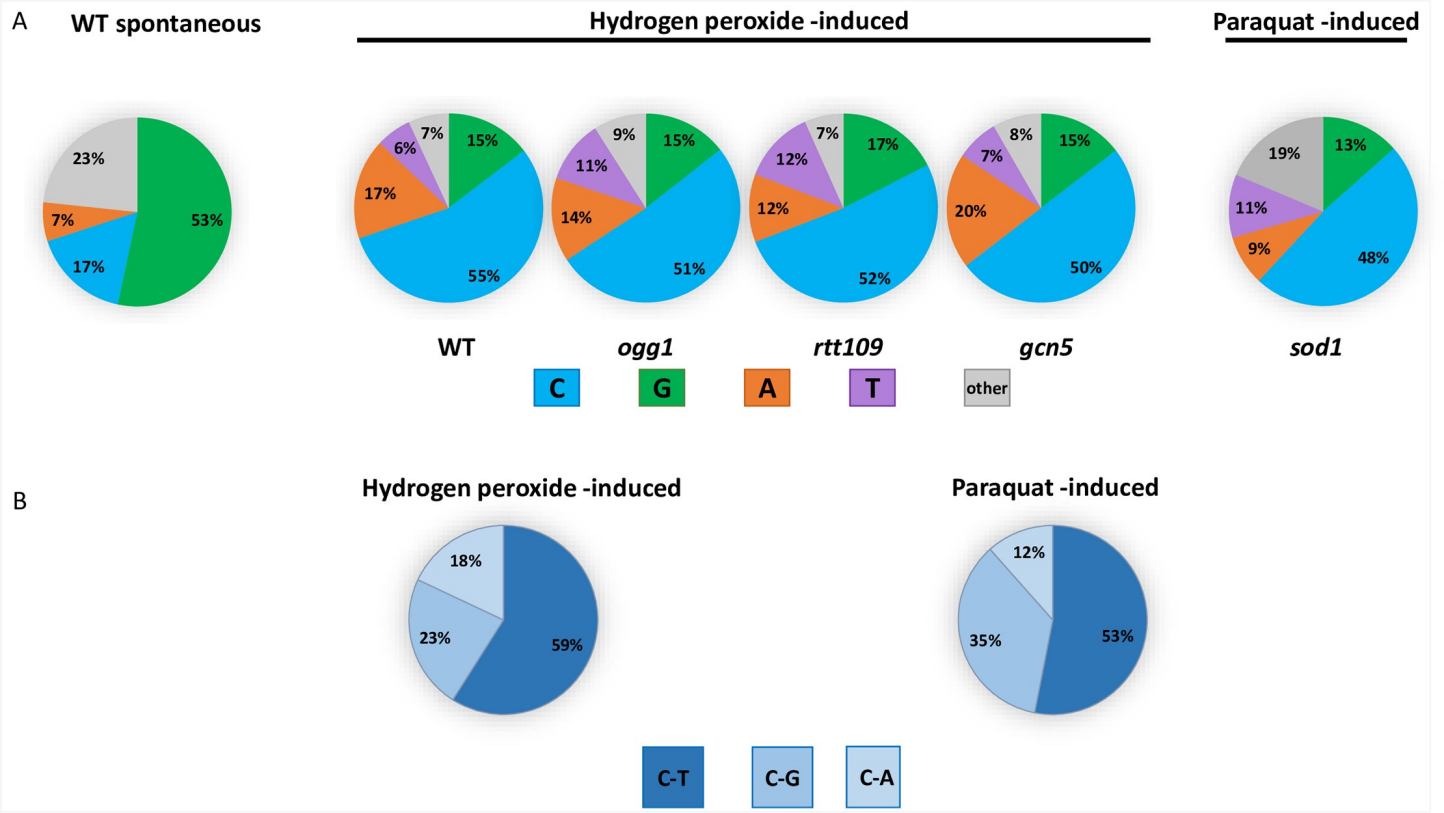
In ssDNA the majority of hydrogen peroxide–and paraquat-induced substitutions in sequenced CanR Red, closely spaced mutants are C to T transitions. A. Spectra of mutations identified by Sanger sequencing of CanR Red isolates in the triple subtelomeric reporter. 131, 141, 146, and 111 hydrogen peroxide–induced mutations had been detected in *wt*, *rtt109*, *gcn5*, and *ogg1* strains, respectively. The differences in spectra of mutations for each mutant background compared to *wt* are not statistically significant by pairwise chi-squared tests (*P* = 0.4, 0.5, and 0.6 for *rtt109*, *gcn5*, and *ogg1* strains, respectively). The difference between spontaneous and hydrogen peroxide–induced spectra of mutations in *wt* strains is significant, *P* < 0.0001. See also S1 Data. B. C to T transitions constitute the majority of mutations at Cs. Left panel: combined data for all of the hydrogen peroxide–induced substitutions at C in ssDNA in all genetic backgrounds tested; right panel: data for paraquat-induced substitutions in *sod1* strains. See also S1 Data. CanR Red, canavanine-resistant red; ssDNA, single-strand DNA; wt, wild-type.

Analysis of the spectrum of the hydrogen peroxide–induced mutations in the triple reporter of CanR Red isolates derived from *wt*, *ogg1*, *rtt109*, and *gcn5* strains revealed that in stark contrast to data obtained by analysis of spontaneous and hydrogen peroxide–induced mutations in dsDNA, the hydrogen peroxide–induced substitutions in ssDNA occur at cytosines more often than at any other nucleotide (Fig 4A, S5 Table, S1 Data). Almost 60% of hydrogen peroxide–induced substitutions at cytosines in ssDNA are C to T transitions (Fig 4B, S1 Data). These data are in good agreement with our previous study in which we could not directly select for clustered mutations and analyzed the spectrum of hydrogen peroxide–induced mutations in the *CAN1* locus of the ssDNA double (*CAN1-URA3*) reporter in strains with different genetic backgrounds, including the strains with deletions of major ROS-neutralizing enzymes, catalase, and superoxide dismutase. The majority of mutations selected in *cta1* knockout strains following exposure to hydrogen peroxide and in *sod1* strains exposed to paraquat also occurred at cytosines [26]. It has been shown previously that during repair synthesis, translesion synthesis (TLS) polymerase inserts A or C across abasic sites (generated by Ung1 removal of U), which leads to equal numbers of C to T and C to G substitutions, whereas in the absence of Ung1, U efficiently pairs with A and C to T. Only in these strains do the substitutions become the major class of mutations [27]. Notably, deletion of *UNG1* did not increase the proportion of C to T transitions among all mutations at cytosines [26]. These data indicate that cytosine deamination is not the underlying source of oxidative damage-induced mutations.

**Fig 5:**
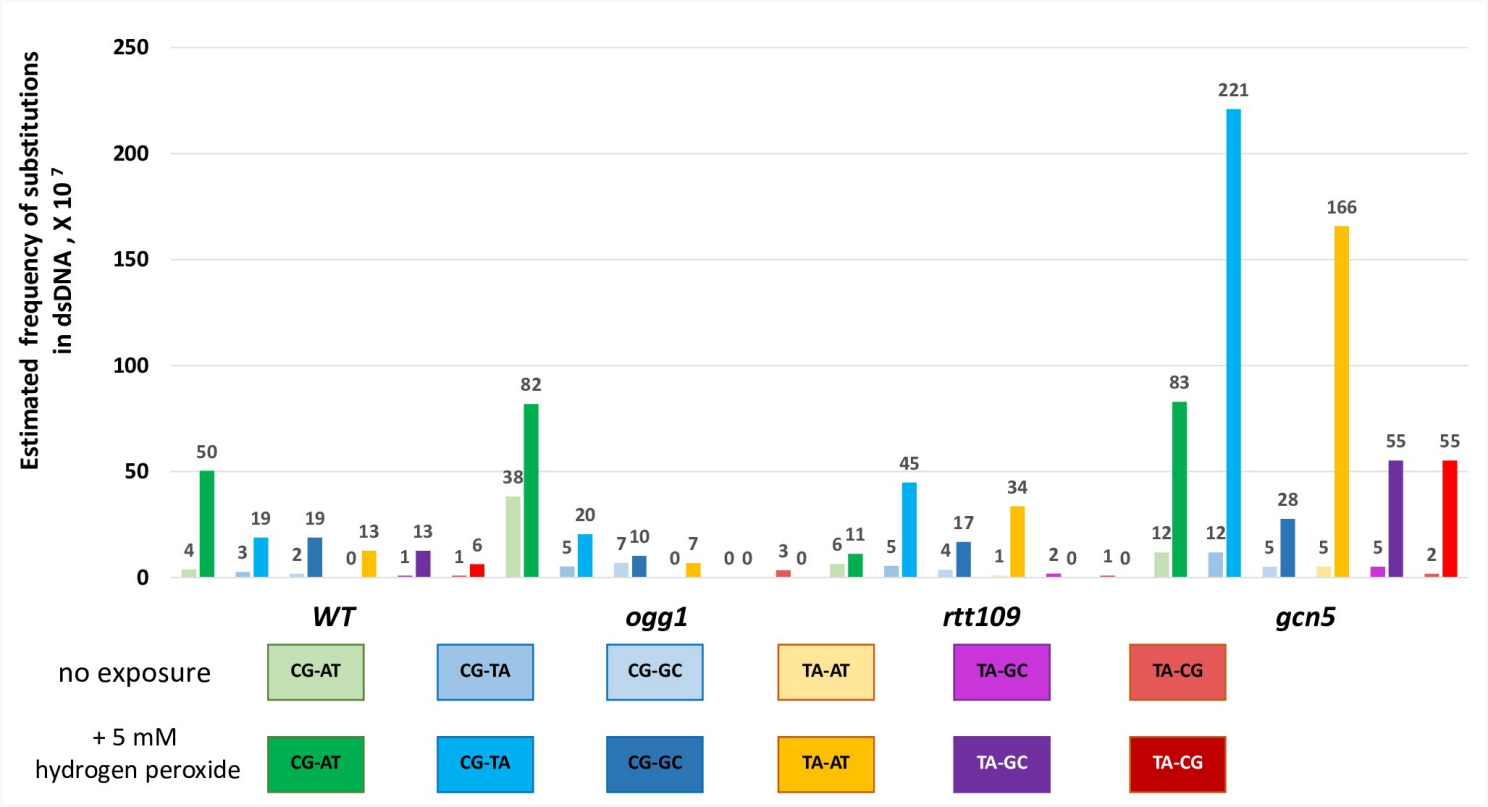
Frequency of mutations at *CAN1* locus in dsDNA. Estimated frequency of spontaneous and hydrogen peroxide–induced CanR mutations in dsDNA. Frequencies of all six possible types of substitutions were calculated by multiplication of the total frequency of the mutations for a specific genotype (S2A Fig) by the percent of the corresponding substitution (Fig 6) as identified by Sanger sequencing of the *CAN1* locus of CanR isolates at midchromosome triple reporter. Note that these are the frequencies of single mutations at *CAN1* locus because in midchromosome reporters, where ssDNA does not form, frequencies of multiple mutations are extremely low. See also S1 Data. CanR, canavanine-resistant; dsDNA, double-stranded DNA.

**Fig 6:**
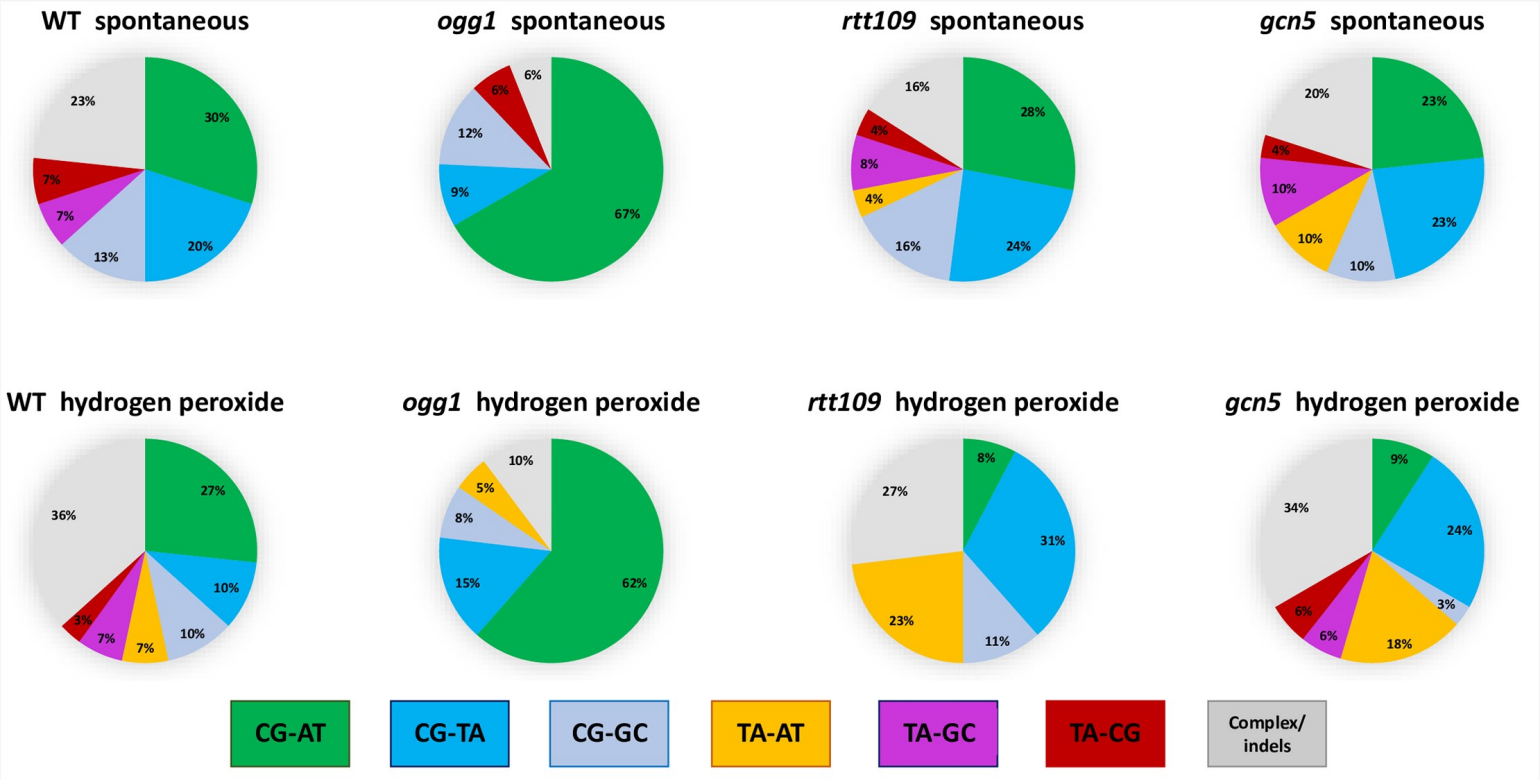
Mutation spectra at *CAN1* locus in dsDNA. Spectra of spontaneous and hydrogen peroxide–induced mutations for all genotypes analyzed. See also S1 Data. dsDNA, double-stranded DNA.

Deletion of *OGG1*, encoding a DNA glycosylase, which initiates repair of 8-oxoG, did not result in either an increased fraction (Fig 4, S1 Data) or in increased frequencies of mutations at guanine in ssDNA (S6 Table), whereas in dsDNA, *ogg1* mutants displayed increases in both spontaneous and hydrogen peroxide–induced mutation frequencies of CanR mutants caused, presumably, by 8-oxoG lesions (GC–TA) in dsDNA (Fig 5, S1 Data). Only in these strains were the majority of substitutions GC to TA transversions (Fig 6, S1 Data). It is important to emphasize that the graphs showing the mutation frequency measurements in ssDNA (Fig 1, Fig 2, right panels of Fig 3A and Fig 3B, and S6 Table), reflect occurrences of closely spaced mutations in at least two adjacent genes (CanR Red colonies), whereas the data reported for the midchromosome reporter display the frequencies and spectra of mutations only in a single gene, *CAN1*, because at that chromosomal location, hypermutable ssDNA does not arise at a permissive temperature. Despite the fact that 8-oxoG is considered the major type of mutagenic oxidative DNA damage, it does not contribute significantly to mutagenesis in ssDNA. Interestingly, the increases in CanR mutation frequency caused by hydrogen peroxide compared to untreated controls were approximately the same in *ogg1* and in *wt* strains (Fig 5, S1 Data). We propose that in dsDNA, Ogg1 glycosylase repairs only endogenously induced lesions at G. One plausible explanation is that an exposure even to a moderately toxic level of hydrogen peroxide exceeds the capacity of Ogg1 to repair the additional damage in dsDNA. Alternatively, hydrogen peroxide may not produce an appropriate substrate for Ogg1 in vivo, suggesting that the major ds- and ssDNA lesion induced by hydrogen peroxide is not 8-oxoG but some other type of DNA modification, which cannot be recognized and repaired by Ogg1.

To ensure that the observed mutation spectra reflect the general mutational consequences of redox stress and are not hydrogen peroxide specific, the spectrum of mutations induced by a different oxidative agent, paraquat, was analyzed. While the precise biochemistry of paraquat-induced oxidative stress is still under debate, it is believed to produce superoxide anions, leading not only to endogenous redox disbalance but also causing mitochondrial dysfunction [38, 39]. Consistent with our previous findings [26], exposure to a moderately toxic dose of paraquat leads to an increase in frequencies of CanR single mutations and closely spaced CanR Red mutants in the strains lacking superoxide dismutase, *sod1* strains (S6 Fig, S1 Data). The mutational spectrum of paraquat-induced mutations was strikingly similar to the spectra of hydrogen peroxide–induced mutations. The majority of substitutions occurred at C (Fig 4A, S1 Data), and more than 50% of them were C to T substitutions (Fig 4B, S1 Data). Such similarity of the spectra of mutations induced by different oxidative agents suggests common underlying DNA lesions and/or mutation-inducing mechanisms.

After elucidating the ssDNA-specific mutation spectrum caused by hydrogen peroxide and paraquat, we sought to determine their mutational signature.

**Fig 7:**
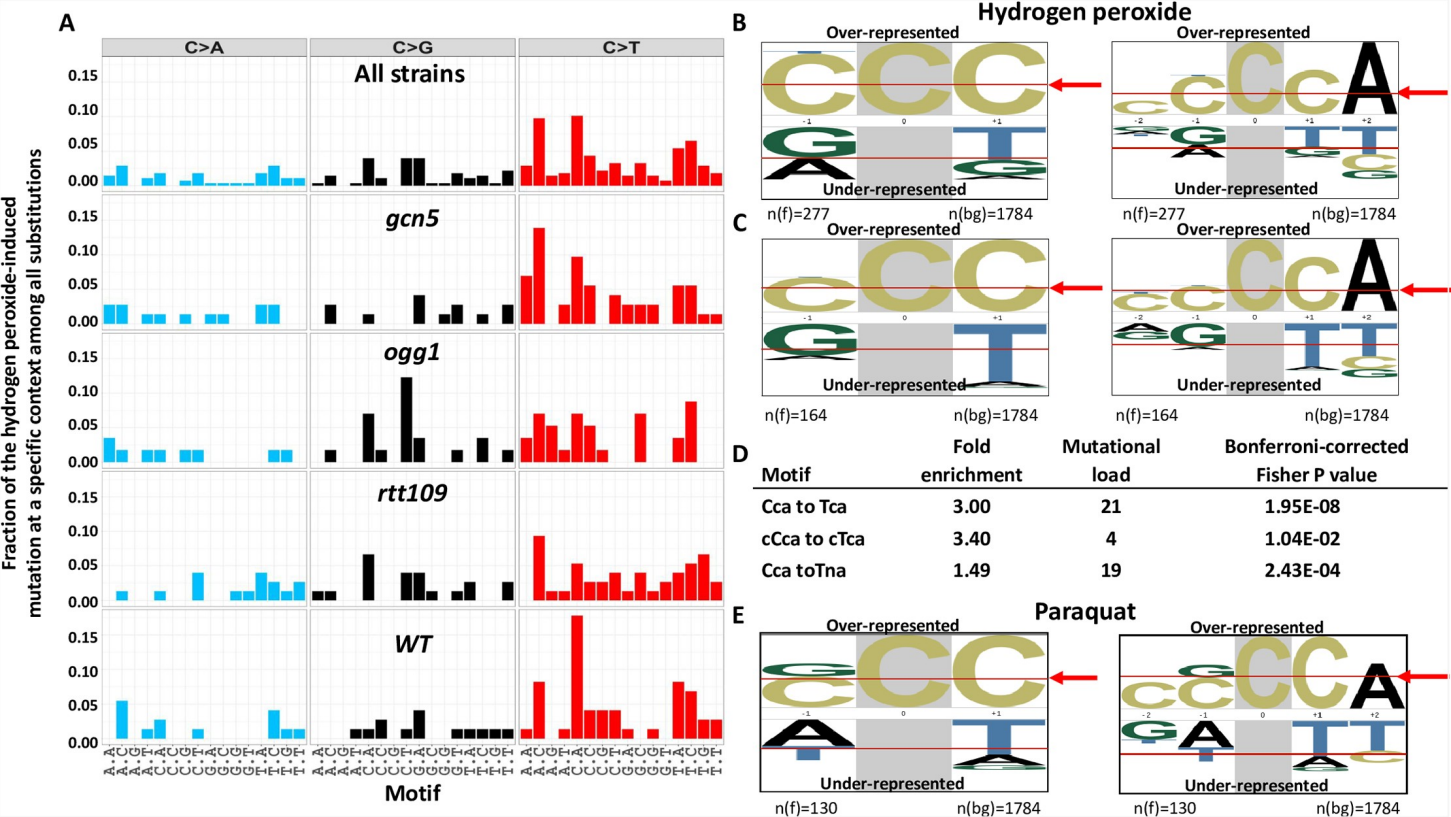
Signature of redox stress–induced DNA damage in ssDNA. A. Fractions of the mutations at C in specific trinucleotide motifs among all of the substitutions detected. B. Overrepresented nucleotides in tri- (left) or pentanucleotide (right) motifs flanking hydrogen peroxide–induced substitutions at C identified by pLogo analysis [40]. The height of each letter indicates the magnitude of over- or underrepresentation. The foreground *n*(fg) = 277 is the number of all substitutions at C. The background *n*(bg) = 1784 is the number of C in the sequence of the subtelomeric triple reporter. The enrichment for the nucleotides above the red arrows is statistically significant, *P* < 0.05. C. Enrichment for the motifs flanking C to T substitutions in hydrogen peroxide–induced mutations (pLogo analysis). The foreground *n*(fg) = 164 are all C to T mutations identified in mutants. D. Fold enrichment for specific motifs (Materials and methods and [13]). The capital C and T indicates the site of C to T mutation; *n* stands for any nucleotide. E. Enrichment for the motifs flanking C substitutions in paraquat-induced mutations in *sod1* strains (pLogo analysis). The foreground *n*(fg) = 130 are all mutations at C identified in mutants. ssDNA, single-strand DNA.

Based on the catalog of all of the mutations in ssDNA (S5 Table), we analyzed the sequence context immediately adjacent to substitutions at cytosines in hydrogen peroxide–induced CanR Red mutants in the wild-type, *ogg1*, *rtt109*, and *gcn5* strains. We noticed that the proportion of substitutions at cytosines considerably varied within different trinucleotide contexts (Fig 7A). However, the increased contribution of a mutation within a specific trinucleotide context to the overall mutation frequencies may reflect a higher occurrence of the specific trinucleotide within the analyzed DNA sequence rather than a higher vulnerability of such DNA context to DNA damage. To distinguish between these possibilities, we first utilized the open source pLogo package [40] that allows estimation of the probability of finding one of the four nucleotides adjacent to the target nucleotide within specific sequence contexts. We found statistically significant overrepresentation of cytosines at the positions −1 and +1 relative to the fixed-position flanking mutated cytosines and an even more significant overrepresentation of adenine at the +2 position (Fig 7B). This overrepresentation was also significant when we analyzed C to T substitutions (Fig 7C). We further confirmed enrichment of the signatures identified by pLogo by our pipeline that is configurable around any signature of choice [41] (Materials and methods) (Fig 7D). When mutations at C in the *CAN1* locus and, independently, in the *ADE2* locus were analyzed for an enrichment against the background of corresponding ORF reference sequences, similar patterns of enrichment were observed, albeit with lower *P* values (S7 Fig). pLogo analysis of paraquat-induced mutations in *sod1* strains uncovered exactly the same pattern of overrepresentation of cytosines one nucleotide to the left and one to the right and adenine in position +2 relative to the cytosines, which among four nucleotides were mutated most frequently by paraquat as well. (Fig 7E). These analyses of mutational signatures induced by different oxidative agents revealed a previously unknown, general signature of redox stress–induced DNA mutagenesis in ssDNA as cCca (upper case C represents the mutated residue) with a preference toward cCca to cTca changes.

### A signature of aging-associated mutagenesis in mitochondrial DNA

**Fig 8:**
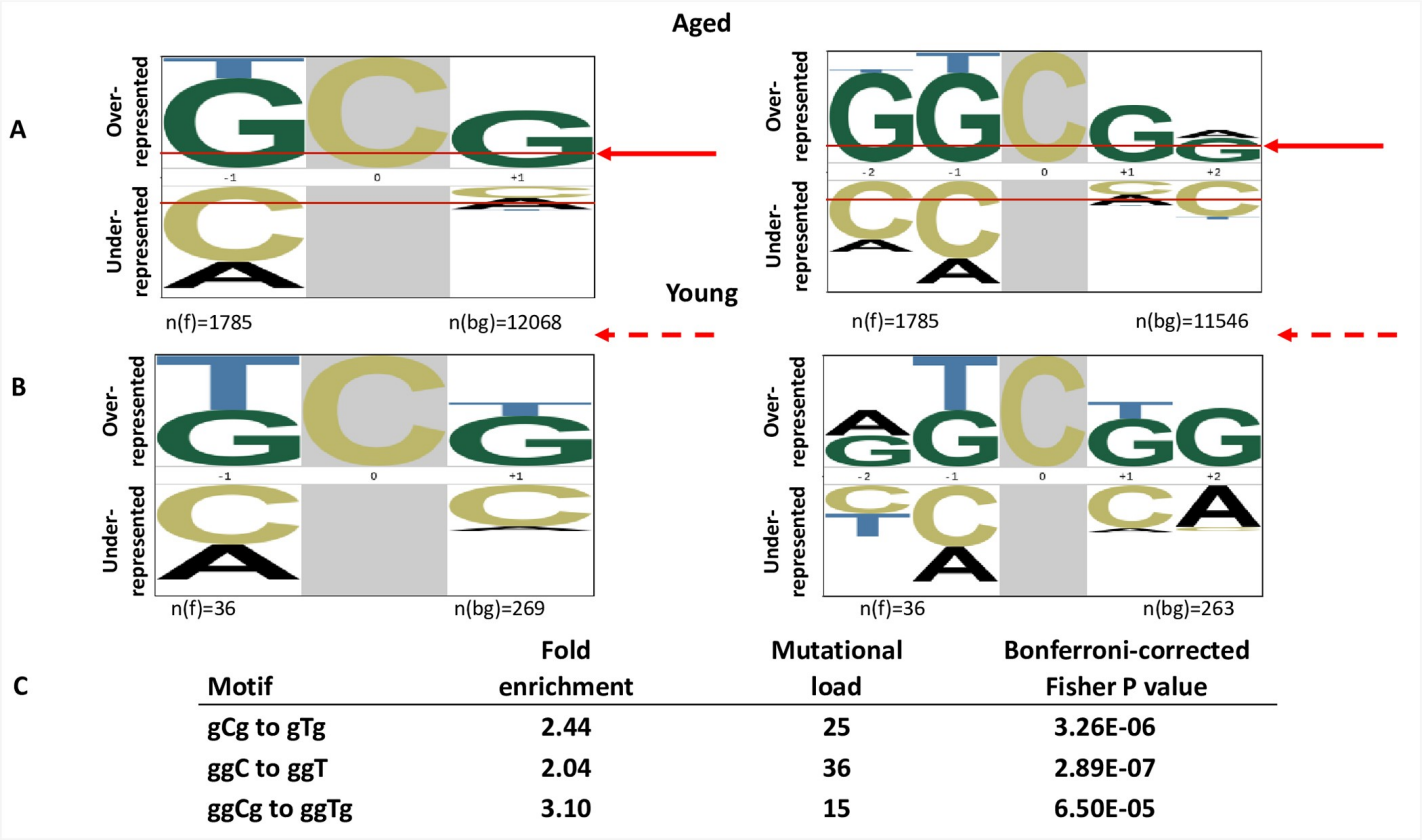
Signature of aging in human mtDNA. A. Overrepresented nucleotides in tri- (left) or pentanucleotide (right) motifs flanking CG to TA transitions, comprising the majority of substitutions in mtDNA in the brains of aged individuals [21] identified by pLogo analysis [40]. The height of each letter indicates the magnitude of over- or underrepresentation. The analysis was based on combined data for C to T substitutions in mitochondria of four aged individuals. The background data set is +/− 20 nucleotides adjacent to the mutation site. The enrichment for the nucleotides above the red arrows is statistically significant, *P* < 0.05. B. pLogo analysis of CG to TA substitutions in mtDNA in the brains of young individuals. The foreground *n*(fg) = 36 is the number of all C to T substitutions in the analyzed mitochondrial sequences. The background data set is +/− 20 nucleotides adjacent to the mutation site. Dashed red arrows above the sequence indicate that none of the adjacent nucleotides are significantly enriched. C. Fold enrichment for specific motifs calculated independently by in-house pipeline (Materials and methods and [13]). The mutated residue and resulting change are capitalized. mtDNA, mitochondrial DNA.

Guided by our finding that the major class of mutations induced by hydrogen peroxide in ssDNA is C to T, we sought to find evidence for redox stress–induced mutagenesis in mitochondrial genomes. Because replication of mitochondrial DNA can be uncoupled, at least temporarily [42], allowing exposure of regions of ssDNA to endogenous DNA-damaging agents, we hypothesized that some features of aging-associated mutagenesis may be similar to the signature of oxidative DNA damage in ssDNA observed in yeast. To test this hypothesis, we analyzed mutation calls obtained by ultrasensitive DNA sequencing reported by Kennedy and colleagues [21] (Materials and methods). This study documented an excess of CG to TA changes over other mutations of GC pairs (CG to AT or to GC) in human mtDNA, which was prevalent in aged (70+ years) brains. Strand bias in CG to TA mutations was observed in the non-D loop region of mtDNA. In reference to the heavy strand of mtDNA, which can be displaced during replication of the leading light strand [43], the bias was in favor of C to T changes, and it was the same as the predominant class of mutations induced by hydrogen peroxide in yeast ssDNA. Therefore, we applied mutation signature analysis to changes in CG pairs. The pLogo analysis revealed ggCg as the predominant mutated motif in all analyzed mtDNA samples combined (Fig 8A) and in each individual sample, with exception of sample UWA 738, which contained a lower number of mutations (117) compared to the other three samples (1,338, 1,147, and 1,170 mutations in each) (S8A Fig). Examination of the C to T substitutions specifically and only in the non-D loop region yielded the same mutational signature (ggCg to ggTg) (S8B Fig). Even though C to T substitutions in mtDNA in young brains were the major class of mutations as they were in aged mtDNA, pLogo analysis did not reveal any significantly overrepresented nucleotides flanking mutated C (Fig 8B), most likely due to an overall low number of mutations detected in young brains. Using our signature-specific configurable pipeline, we further demonstrated that the ggCg to ggTg change is statistically significantly enriched in aged mtDNA (Fig 8C). This signature was different from the signature we found for hydrogen peroxide–induced mutations in ssDNA in yeast, suggesting that each mutational signature reflects complex organism- and/or localization-specific (nuclear versus mitochondrial) metabolic processing of DNA lesions rather than revealing only the vulnerability of a specific nucleotide context to redox stress–induced DNA damage.

### Evidence for redox stress–related mutation signatures in human cancers

**Fig 9:**
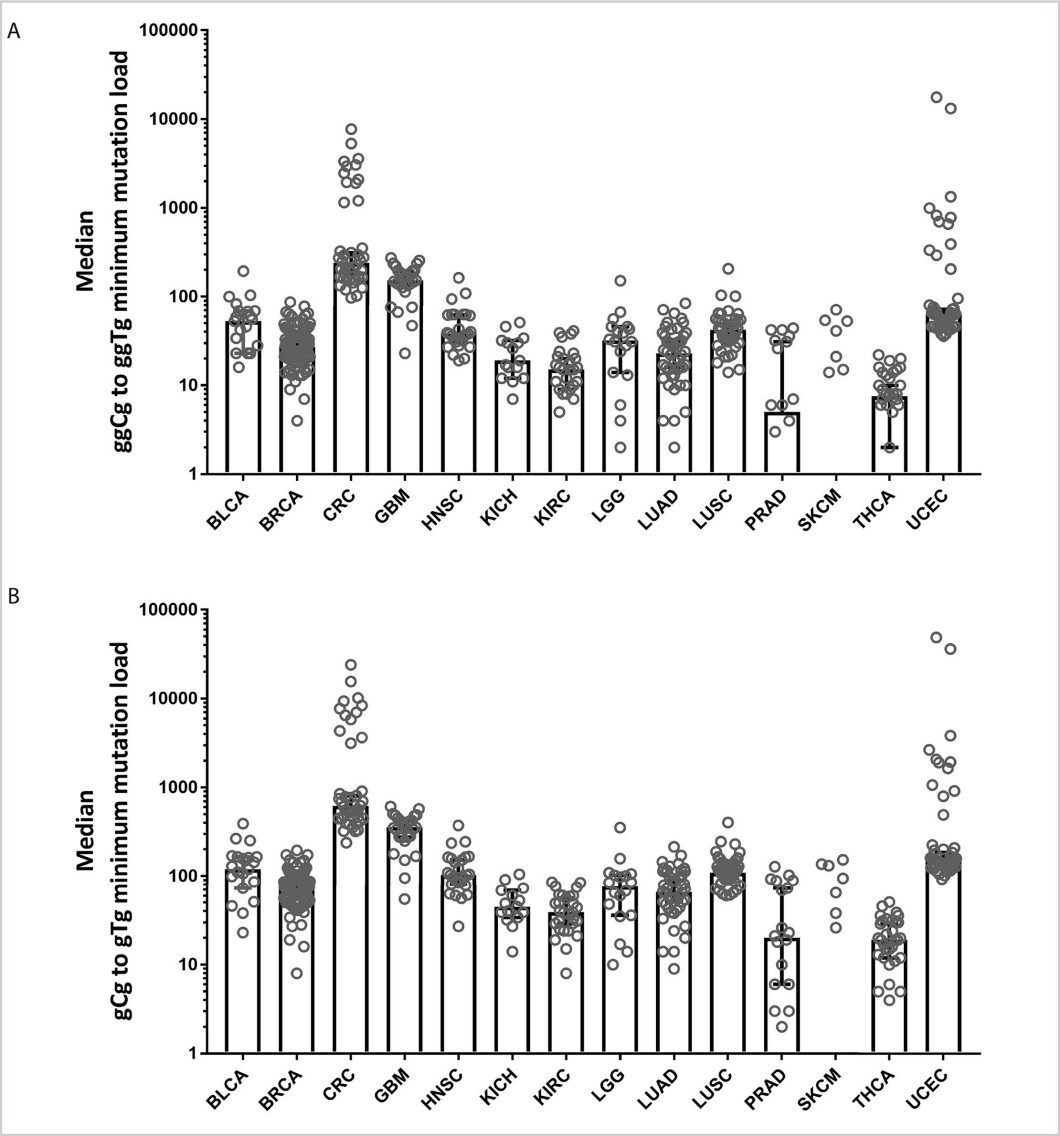
Evidence for redox stress–related signatures in human cancers. Medians of minimum estimates of mutation load assigned to ggCg to ggTg (A) and gCg to gTg (B) signatures are depicted as bars. Gray circles represent minimum mutation load for each individual cancer genome sample. Whiskers of the bars represent standard errors. See also S1 Data. BLCA, bladder urothelial carcinoma; BLCA, breast invasive carcinoma; CRC, colorectal carcinoma; GBM, glioblastoma multiforme; HNSC, head and neck squamous cell carcinoma; KICH, kidney chromophobe; KIRC, kidney renal clear cell carcinoma; LGG, brain lower-grade glioma; LUAD, lung adenocarcinoma; LUSC, lung squamous cell carcinoma; PRAD, prostate adenocarcinoma; SKCM, skin cutaneous melanoma; THCA, thyroid carcinoma; UCEC, uterine corpus endometrial carcinoma.

In search for newly discovered redox stress–related signatures, we analyzed mutation calls from 505 whole genome–sequenced human cancers [44]. Our pursuit of the yeast signature of redox stress in cancers did not produce robust results: only a few individual cancer genomes had slightly increased mutational load of Cca to Tca changes; however, we detected mitochondrial redox stress gCg to gTg and ggCg to ggTg signatures, as manifested by increased mutation loads associated with these motifs, in all cancer types tested (Fig 9, S7 Table)

Closely spaced, clustered mutations in cancer genomes are likely caused by damage of persistent ssDNA. By utilizing the ssDNA reporter system in this study, we modeled these cancer-specific mutagenic events. Also, strand bias in the mutations detected in mtDNA in aging brain suggested that the clustered mutations accumulated in mitochondria in ssDNA. Therefore, we further analyzed cancer genomes for the same signatures in regions with clustered mutations. Analyzing all cancer samples within a cohort, we detected a significant enrichment for gCg-to-T and ggCg-to-T mutations in clusters across various cancer types (S8 Table). These data imply that oxidative damage contributes to C→T mutations in ssDNA in cancers.

The gCg-to-T mutation signature has previously been found to also be enriched in endometrial and colorectal cancers due to the erroneous bypass of methylated cytosines by mutated replicative polymerases [45]. In agreement with this, we found both gCg-to-T and ggCt-to-T mutations to be prevalent in uterine corpus endometrial carcinoma (UCEC) and colorectal carcinoma (CRC) samples. To rule out the possibility that these mutation signatures were due to the activity of defective DNA polymerases, we further measured enrichment of the DNA polymerase mutation signatures (tCg to tTg, tCt to tAt, and tTt to tGt), which are different from redox stress signatures, across all cancer types and determined their correlation with the ggCg to ggTg mutation signature, which is more specific for oxidative damage than the gCg to gTg signature. The replicative polymerase signatures were found to correlate with each other in UCEC, CRC, and lung adenocarcinoma (LUAD) samples, and these signatures were also found to correlate with ggCg to ggTg signature across the UCEC and LUAD samples. However, we did not detect a statistically significant correlation between the ggCg to ggTg redox stress signature and all the replicative polymerase-associated signatures in the other cancer types (S9 Table). Moreover, the gCg-to-gTg mutations in endometrial cancers have previously been shown to occur more often on the lagging strand than on the leading strand during DNA replication [45]. Analysis of the gCg-to-gTg as well as the ggCg-to-ggTg mutations on the leading or lagging strands lead to a conclusion that in cancers other than UCEC and CRC, these mutations do not demonstrate a replication bias (S10 Table). These observations indicate that the mechanisms underlying the ggCg-to-ggTg mutation signatures are independent of the replicative DNA polymerase-induced mutations. We propose that these signatures are due to oxidative damage of cytosines.

## Discussion

The discovery of mutational clusters in various cancer genomes has led to the realization that the hypermutability of ssDNA is relevant to cancer etiology [46]. By employing a yeast reporter system for detecting mutation clusters, we found a characteristic mutation spectrum and mutational signatures for redox stress in ssDNA. These results have important implications for understanding mutagenesis in nuclear and in mitochondrial DNA as well as for age-related mutagenesis. If redox stress contributes to genome destabilization, the signature of oxidative damage in ssDNA should be discernable in cancer genomes and other pathologies. Diagnostic detection of such a signature could have important clinical implications in making therapeutic decisions based on a patient’s specific cancer genotype(s). However, prior to this study, the mutational signature of redox stress in ssDNA had not been reported.

### The signature of oxidative damage in ssDNA and the signature of aging in mtDNA are centered around predominant C to T substitutions

By utilizing an improved reporter system, we overcame the obstacles in elucidating the mutational signature, such as low mutagenicity of oxidative agents and decreased density of clustered mutations in ssDNA. We documented a preponderance of C to T (not G to T) substitutions resulting from ROS-induced lesions at C in ssDNA in yeast. Our finding that acetyltransferase Gcn5 and, to a lesser extent, its functional homolog Rtt109 protect ssDNA from mutagenesis by oxidative damage provides a sensitive tool for characterizing hydrogen peroxide–induced mutagenesis in ssDNA. These acetyltransferases are engaged in the process of replication-coupled nucleosome assembly [47, 48] and repair of replication-generated DNA double-strand breaks [31, 32]. The increase in mutation density and in the number of closely spaced mutations located within 147 bp from each other (the length of DNA wrapped around a nucleosome) in ssDNA in the absence of Rtt109 and Gcn5 may also provide evidence for a recent, unexpected finding that histones can remain associated with ssDNA following DNA resection and that the ssDNA–histone complexes are stable and can activate chromatin remodeling [49]. Studies of homologous recombination repair in yeast suggest that the presence of histones on ssDNA is crucial for promoting DSB repair [50]. Further studies of the role of chromatin status regulated by Rtt109 and Gcn5 are necessary for understanding their role in protection of ssDNA from oxidative DNA damage.

Importantly, our ssDNA reporter system allowed for unequivocal identification of cytosine as a nucleotide at which redox stress–induced mutations occur most often. In contrast, analysis of the mutations in dsDNA does not reveal this information, since the damage could occur in either strand of DNA. The finding that cytosine is the most mutagenic of the nucleotides in ssDNA is consistent with observations that in mtDNA of aging human brains [21] and in flies [22], CG to TA substitutions were the major class of point mutations. Moreover, in aged human brains, there was a preference of C to T over G to A changes (strand bias) in reference to the mtDNA heavy strand despite the fact that this strand is G-rich. We propose that the lesions are occurring at Cs in the mtDNA heavy strand when DNA replication starts at the light chain, temporarily making the displaced, heavy strand more vulnerable to damage.

### The signature of redox stress in nuclear and mitochondrial ssDNA as a tool for uncovering molecular mechanisms of genome destabilization in pathological conditions and in aging

Information about the signature of redox stress in ssDNA and the signature of aging in mitochondrial DNA can open an avenue in research aimed at understanding the consequences of redox stress in biological systems. This knowledge can be used to detect footprints of oxidative stress as well as for revealing specific processes and mechanisms depositing such signatures in genomes. These two analytical approaches can be facilitated by recently developed, vast repositories of human genomics data.

Taking into consideration the significance of redox stress as a hallmark of cancer, it seems important to discern to what degree the genomic instability of a specific cancer type can be ascribed to redox disbalances. Several mutational signatures already found in human cancers were attributed to malfunction of specific enzymes with defined target sequences, such as APOBEC hypermutation signatures in human cancers [51], or ones caused by strong mutagens, such as UV light [52], aristolochic acid [53], and many others (reviewed in [54]). The signatures of redox stress found in our study likely reflect the interplay of several processes, such as the context-specific deposition of oxidative stress–induced lesions, lesion bypass by DNA polymerases, and cell death caused by such lesions. The signatures highlighted by our study have a common feature with several established cancer signatures in being centered around C-to-T substitutions. Such overlap may potentially obscure the contribution of redox stress to the mutation signature in a tumor. Nevertheless, we were able to find redox stress–related signatures in several cancers (Fig 9) as well as significant enrichment for fairly strict ggCg-to-ggTg mutational signature in cancers with high frequency of clustered mutations, i.e., enriched with persistent ssDNA. Even though these signatures could have been attributed to a subset of previously reported, less stringent mutational signatures of the replicative DNA polymerase errors in UCEC and CRC samples, our analysis suggests that the oxidative signature is distinct and different because we were not able to detect the replication bias for these mutations. It is important to emphasize that these findings confirm that the knowledge-based signatures we discovered are present in cancer genomes and can be distinguished from other types of the mutational signatures. If such signatures were to be deciphered by discovery-based biocomputational approaches it would have been impossible to attribute them to any known mutational/oncogenic processes. Further studies of the consequences of exposure of human cells to oxidative agents and more sensitive biostatistical tools will improve the probability of a successful search for the mutational signatures in future.

Redox stress and inflammation are often cited as exacerbating factors in neurodegenerative diseases (reviewed in [55, 56]). If the mutational signature of aging in mtDNA mirrors, at least partially, the footprint of oxidative stress, detection of an increased mutational load at a defined and finite number of motifs within the relatively small human mitochondrial genome could serve as a biomarker for increased risk of development of such disease(s) or as a marker of acute exposure to environmental oxidants.

### What are the mechanisms underlying the mutational signature of redox stress in ssDNA?

An important insight in understanding the molecular mechanisms of redox stress in vivo can be inferred based on the data obtained in this study and our previous studies (26), which argue that 8-oxoG is not the major mutagenic lesion in ssDNA. Two lines of evidence provided by our experiments—predominance of C to T mutations in ssDNA in yeast and human mtDNA in aged individuals as well as mutation strand bias indicating that mutagenic lesions at cytosines occur in a strand persisting as ssDNA—suggest that the signature of aging can be attributed to mechanisms similar to or overlapping with mechanisms of accumulation of mutagenic oxidative damage in ssDNA.

While many features of mechanisms resulting in ROS-induced mutagenesis in ssDNA in yeast and in mtDNA in aging human brains may be quite different, the preponderance of C to T changes in both biological contexts is striking. This similarity of these mutation spectra suggests that the prevailing mutagenic lesion(s) are also similar. So far, several cytosine modifications are known to be mutagenic in ss- and dsDNA. Cytosine oxidation and deamination produce 5-hydroxyuracil and uracil glycol, which has been shown to cause C to T transitions when ssDNA oligos containing these modified bases are transfected into *Escherichia coli* [57]. 5-hydroxycytosine is also mutagenic when bypassed by DNA polymerases [58, 59]. 5-chlorocytosine, a known human inflammation biomarker, causes C to T transitions with high frequencies when electroporated into *E*. *coli* in a ssDNA reporter system [60]. However, it is still unclear which of these lesions or, possibly, which combination of them could be causing C to T mutations in ssDNA exposed to ROS. Our finding that C to T substitutions occur in a specific DNA context in ssDNA (cCca) may facilitate biochemical studies aimed at precise identification of the redox stress–induced lesions in DNA.

## Materials and methods

### Strains construction

All yeast strains used in this study are derivative of diploids JFS1427, JFS1429 harboring subtelomeric triple reporter and JFS1432, JFS1433 with midchromosome triple reporter. Triple subtelomeric reporter–containing parental diploid strains were made by crossing *cdc13-1ts* and *CDC13* derivatives of the CG379 (*MATα his7-2 leu2-3*,*112 trp1-289*) [36] strains in which the *ADE2*, *CAN1*, and *URA3* genes were deleted from their native locations and reintroduced into the left subtelomeric region of Chromosome V [27]. Strains carrying midchromosome reporter were also constructed by crossing of *cdc13-1ts* and *CDC13* parents originated from YSR128 strain (*MATα ura3Δ can1Δ ade2Δ leu2-3*,*112 trp1-289 lys2*:*ADE2-URA3-CAN1*) in which *CAN1*, *URA3*, and *ADE2* genes were inserted into the *LYS2* locus. The disruption of the genes of interest had been performed by transformation of diploid strains with the PCR fragments containing antibiotic resistance genes flanked by upstream and downstream sequences of the corresponding genes. Deletions were confirmed by PCR. To avoid accumulation of unselected mutations and modifications, all haploid strains subjected to mutation frequency measurements were obtained in 6–12 fresh haploid isolates from meiotic tetrad dissection of heterozygous diploids.

### Genetic procedures and media

Genetic manipulations with yeast (transformation, tetrad analysis, etc.) were carried out as described previously [61]. Strains were grown at 30°C, *cdc13-1ts* mutants were grown at room temperature (23°C), or at 37°C for induction of telomere uncapping.

### Measurements of the frequencies of closely spaced mutations at triple subtelomeric reporter (CanR Red) and mutations in CAN1 (CanR)

Measurements of spontaneous and DNA-damaging agents–induced frequencies of mutations at subtelomeric reporter were performed as following. Mutant haploid spores of interest and *wt* controls dissected simultaneously from the parental heterozygous diploid were incubated at 23°C for 72 hours in rich medium. Following 10-fold dilution in rich medium, cultures were incubated in a shaker at 37°C for 6 hours, quickly washed, and split into two cultures, one of which was exposed to DNA-damaging agent; another was mock treated and used for control. To estimate the frequency of hydrogen peroxide–induced mutations, cells were exposed to 5 mM or 10 mM hydrogen peroxide in water for 2 hours at 37°C. For measuring of MMS-induced frequencies, the cells were exposed to 11.8 mM MMS in phosphate buffer saline for 30 minutes at 37°C. The reaction was terminated by incubation with sodium thiosulfate. For UV exposure, cell suspensions in water were poured to Petri dishes and exposed to 40 J/m2 UV in the UV cross-linker (Stratalinker 2400). After the exposure cells were washed, and for selection of *CAN1 ADE2* multiple mutants, they were plated onto synthetic complete medium lacking arginine, containing a reduced (20 mg/l) amount of adenine, and supplemented with 60 mg/l canavanine. A diluted suspension was plated onto synthetic complete medium without arginine for calculation of total number of cells in culture. After 1 week of incubation at 23°C, plates were moved to 4°C and incubated for an additional week. CanR Red mutation frequency was calculated as a ratio of CanR Red colonies to total number of cells in culture. Survival was calculated as the ratio of total number of cells in exposed culture divided to the number of cells in corresponding nonexposed culture. Measurements of single-mutation frequencies at *CAN1* locus at subtelomeric and at midchromosome reporter were performed as described above, with the only difference being that frequency of mutation was calculated as ratio of CanR cells to all viable cells in culture. In experiments involving *sod1* mutants, the media was supplemented with L-methionine and L-lysine to the final concentrations of 200 mg/ml and 300 mg/ml, respectively.

### Measurements of the frequencies of CanR mutations at triple midchromosome reporter

Mutant haploid spores of interest and *wt* controls dissected simultaneously from the parental heterozygous diploid were incubated at 23°C for 72 hours in rich medium. Cultures were diluted into fresh rich medium and incubated at 37°C for 6 hours. Each culture was split into two and either exposed or mock exposed to 5 mM hydrogen peroxide for 2 hours. Cells from the cultures were plated on synthetic medium lacking arginine and supplemented with 60 mg/ml of canavanine and, after appropriate dilutions, onto synthetic medium lacking arginine without canavanine. Frequency of mutations was calculated as the ratio of CanR cells in cultures to the total number of cells.

### Identification of mutations in ssDNA and dsDNA reporters

CanR Red colonies were isolated from the canavanine-containing plates, streaked for single colonies, and the genotypes were rechecked by replica plating onto complete synthetic medium without adenine and onto selective medium without arginine, supplemented with canavanine. An individual colony from each streaking was used for genomic DNA isolation with MasterPure Yeast DNA Purification kit (Epicentre). *CAN1*, *URA3*, and *ADE2* ORFs as well as 3′- and 5′ flanking −*LYS2* sequences were amplified by PCR using primers listed in S7 Table. Sequencing of these PCR products was performed by Eton Biosciences and GENEWIZ using the primers listed in S11 Table. Mutations were identified using Seqman software (DNASTAR, Madison, WI) and graphed using Excel (Microsoft, Redmond, WA). Identification of CanR mutations in midchromosome reporter was performed with the same primers that were used for sequencing of the *CAN1* locus in triple subtelomeric reporter (S11 Table).

### Analysis of oxidative damage–associated mutation signatures

The mutation spectra of all changes at cytosines were analyzed using the Bioconductor package Somatic Signatures [62]. The function plotMutationSpectrum() was used to generate graphical outputs of the mutation spectrum.

pLogo was used to identify nucleotides in the foreground comprised of the mutated residue and the −2, −1 and +1, +2 positions that were statistically over- or underrepresented relative to the background sequence. The background sequence was set as the triple-reporter sequence for yeast mutation data and mitochondrial genome sequence for analyzing the mitochondrial mutations in humans. C→T substitutions were analyzed, and the mutated C was set to position 0. The reverse complements were also used in the analysis, except for analysis involving ssDNA of the subtelomeric triple reporter. Nucleotides represented above the red horizontal axis were statistically overrepresented in the foreground with *P* values < 0.05. The height of each residue in the output represents the degree of over/underrepresentation in the foreground [40].

The enrichment of oxidative damage–associated mutation signatures and minimum mutation loads in yeast and the aging-associated mutation signatures in mitochondrial genomes were calculated similar to Saini and colleagues and Chan and colleagues [41] [51]. In short, the number of C→T changes within a tri- or tetranucleotide motif were compared to the total number of C→T changes in the sequenced regions. This ratio was further compared to the number of incidences of the motif versus the number of cytosines present in the +/− 20 nucleotide flanks of the mutated motifs. For each motif, the reverse complement was also used in the calculations. An example of the enrichment calculation is provided below:

$${Enrichment}_{\left(Cca\to Tca\right)}=\frac{{mutations}_{\left(Cca\to Tca\right)}\times {context}_{\left(c\right)}}{{mutations}_{\left(C\to T\right)}\times {context}_{\left(cca\right)}}$$

To statistically determine the overrepresentation of the mutation signatures, a one-sided Fisher’s exact test was used. To correct for multiple testing, Bonferroni-correction was applied. The minimum mutation loads attributable to a signature were calculated for samples with enrichment value > 1 and the Bonferroni-corrected *P* value < 0.05. For samples that had enrichment < 1 or the corrected *P* values > 0.05, minimum mutation loads were attributed a value of 0. An example of the calculation for minimum mutation load is provided below:

$${MutLoad}_{\left(Cca\to Tca\right)}=\frac{{mutations}_{\left(Cca\to Tca\right)}\times ({Enrichment}_{\left(Cca\to Tca\right)}-1)}{{Enrichment}_{\left(Cca\to Tca\right)}}$$

### Analysis of replicative strand bias for mutation signatures

The annotations for left- or right-replicating regions of the genome were obtained from Asymtools [63]. Bedtools intersect was used to identify ggCg to ggTg mutations within left- or right-replicating regions of the genome. Mutations in cytosines in left-replicating regions and mutations in guanines in right-replicating regions were annotated as mutations in cytosines on the lagging strand. A one-sided binomial test was utilized to identify if the bias in mutations to the left- or right-replicating regions was statistically significant. The Benjamini–Hochberg correction for multiple testing was applied to obtain corrected *P* values.

### Determination of correlation between mutation signatures

Pearson correlation coefficient was calculated for the enrichment values of the ggCg to ggTg mutation signature versus the mutation signatures characteristic of replicative polymerases. The resulting *P* values were corrected for multiple testing using Benjamini–Hochberg correction.

### Quantification and statistical analysis

Fisher’s exact test, chi-squared, and Mann–Whitney tests were used for statistical analysis of the data. Choices of one-sided or two-sided versions are indicated, and exact *P* values are shown with every analysis. Unless stated otherwise, the whiskers above and below the bars in all the graphs represent 95% confidence limits of the median of corresponding values.

## Supporting information

S1 Fig Modulation of chromatin status of the cells causes changes and frequencies of mutations in ssDNA. A. Exposure to 50 mM NAM increases frequency of spontaneous but not hydrogen peroxide–induced mutations. All experiments were carried out as described in Materials and methods. Cultures inoculated from independent colonies were incubated with or without NAM in rich medium for 72 hours at room temperature. Cultures were diluted into fresh rich medium and incubated at 37°C for 6 hours. Each culture was split in two and either exposed or mock exposed to 5 mM hydrogen peroxide for 2 hours. Cells from the cultures were plated on synthetic medium with decreased amount of adenine lacking arginine and supplemented with 60 mg/l of canavanine and, after appropriate dilutions, onto synthetic medium lacking arginine without canavanine. Frequency of mutations were calculated as the ratio of CanR Red cells to the total number of cells in cultures. Mutation frequencies for six to 12 independent cultures were measured in each experiment. B. Hst3 or Hst4 histone deacetylases are redundant in protection of ssDNA from oxidative damage; double mutant exhibits increased mutation frequencies and cell killing. Independent spores of each genotype were inoculated into rich medium and incubated at room temperature for 72 hours. Cultures were diluted into fresh rich medium and incubated at 37°C. Aliquots of cultures were taken after before the beginning of incubation at 37°C and after 1, 2.5, and 5 hours of incubation; cells were plated onto complete synthetic medium lacking arginine and supplemented with 60 mg/l of canavanine and, after appropriate dilution, plated onto complete synthetic medium. Mean frequency of CanR and standard errors are shown. See also S1 Data. CanR Red, canavanine-resistant red; H_2_O_2_, hydrogen peroxide; NAM, nicotinamide; ssDNA, single-strand DNA.
(PPTX)

S2 Fig Hydrogen peroxide–induced mutagenesis in dsDNA and ssDNA. A. Exposure to hydrogen peroxide increases mutation frequencies in dsDNA. Eight to 16 independent, freshly dissected spores of each genotype harboring midchromosome triple reporter were inoculated into rich medium and incubated at 23°C for 72 hours. Cultures were diluted into fresh rich medium and incubated at 37°C for 6 hours. Each culture was split in two and either exposed or mock exposed to 10 mM hydrogen peroxide for 2 hours. Cells from the cultures were plated on synthetic medium lacking arginine and supplemented with 60 mg/l of canavanine and, after appropriate dilutions, onto synthetic medium lacking arginine without canavanine. Frequencies of mutations were calculated as the ratio of CanR cells in cultures to the total number of cells.
B. Exposure to hydrogen peroxide increases frequencies of the clustered mutation in ssDNA. Eight to 16 independent, freshly dissected spores of each genotype harboring subtelomeric triple reporter were inoculated into rich medium and incubated at 23°C for 72 hours. Cultures were diluted into fresh rich medium and incubated at 37°C for 6 hours. Each culture was split into two and either exposed or mock exposed to 5 mM hydrogen peroxide for 2 hours. Cells from the cultures were plated on synthetic medium with decreased amount of adenine, lacking arginine and supplemented with 60 mg/ml of canavanine and, after appropriate dilutions, onto synthetic medium lacking arginine, without canavanine. Frequencies of mutations were calculated as the ratio of CanR Red cells to the total number of cells in culture. Whiskers represent 95% confidence interval for median frequency. See also S1 Data. CanR Red, canavanine-resistant red; dsDNA, double-stranded DNA; ssDNA, single-strand DNA.
(PPTX)

S3 Fig Density and distribution of adjacent mutations in the acetyltransferases mutant strains differs from density and distribution of mutations in the strains with nonperturbed chromatin status. A. Fraction of nonselected mutations in CanR Red isolates in *rtt109* and *gcn5* strains is larger than in *wt* and *ogg1* strains. Schematic presentation of the proportion of mutants with 2, 3, 4, 5, and 7 mutations identified by Sanger sequencing of the subtelomeric reporter sequence (Fig 1A) for corresponding number (*n*) of CanR Red mutants of each genotype. Fraction of mutants with nonselected mutations (3 and more) for *rtt109* and *gcn5* strains is significantly higher than that for *wt* and *ogg1* strains (*P* = 0.04 for one-sided Fisher exact test). B. Number of adjacent mutations with the certain distance between them as a fraction of total number of adjacent mutations. To compare the distribution of closely spaced mutations in *rtt109* and *gcn5* histone acetylase mutants with the distribution in the strains with nonperturbed chromatin status (*wt* and *ogg1*), the data for corresponding groups were combined and presented as percent of mutations with distance between them within the bins of 1 to 400 bp, 401 to 800 bp, 801 to 1,200 bp, and 1,201 to 1,600 bp. Distribution of distances between the groups was significantly different (chi-squared *P* = 0.04). C. Distribution of the distances between hydrogen peroxide-induced, adjacent mutations in *CAN1* and *ADE2* loci in triple subtelomeric reporter of CanR Red isolates. Each sphere represents the distance between two adjacent mutations identified by Sanger sequencing of independent CanR Red isolates in wild-type and mutant strains. See also S1 Data. CanR Red, canavanine-resistant red; wt, wild-type.
(PPTX)

S4 Fig Maps of the mutations of all the sequenced CanR Red isolates in the triple-reporter sequence. The map of the mutations in CanR Red isolates in wt (A), *rtt109* (B), and *gcn5* (C) strains. Reference sequence is presented schematically to the right of the map of mutations in *wt* strains. Red arrows highlight pairs of mutations that are less than 147 bp apart. See also S5 Table. CanR Red, canavanine-resistant red; wt, wild-type.
(PPTX)

S5 Fig Exposure to a moderately toxic dose of UV irradiation leads to a higher frequency of CanR Red mutations in *wt* compared to *rtt109* cells. Eight freshly dissected spores of each genotype were inoculated into rich medium and incubated at 23°C for 72 hours. Cultures were diluted into fresh rich medium and incubated at 37°C for 6 hours. Each culture was split in two and either exposed or mock exposed to UV at 40 J/m^2^ in Stratalinker 2400 UV Crosslinker (Stratagene). Cells were plated on synthetic medium with decreased amount of adenine, lacking arginine and supplemented with 60 mg/l of canavanine and, after appropriate dilutions, onto synthetic medium lacking arginine, without canavanine. Frequencies of mutations were calculated as the ratio of CanR Red cells to the total number of cells in cultures. Frequencies of mutations in *wt* strain were significantly higher than in *rtt109* strain (*P* = 0.01, Mann–Whitney test). See also S1 Data. CanR Red, canavanine-resistant red; UV, ultraviolet irradiation; wt, wild-type.
(PPTX)

S6 Fig **Exposure to paraquat leads to significant increase in frequencies of CanR single (A) and closely spaced (B) CanR Red mutants in *sod1* strains.** Four wild-type and 12 *sod1* freshly dissected spores of each genotype were inoculated into rich medium and incubated at 23°C for 72 hours. Cultures were diluted into fresh rich medium and incubated at 37°C for 6 hours. Each culture was split into two and either exposed or mock exposed to 50 micromolar paraquat in water for 1.5 hours. Cells from the cultures were plated on synthetic medium with decreased amount of adenine, lacking arginine and supplemented with 60 mg/ml of canavanine, methionine, and lysine (Material and methods) and, after appropriate dilutions, onto synthetic medium lacking arginine without canavanine and supplemented with methionine and lysine. Frequencies of mutations were calculated as the ratio of CanR or CanR Red cells s to the total number of cells in culture. *P* values were determined by the Mann–Whitney test. See also S1 Data. CanR Red, canavanine-resistant red.
(PPTX)

S7 Fig Validation of the mutational signature of oxidative stress–induced mutations in ssDNA. pLogo analysis for the motif’s enrichment of a subset of mutations at C in *CAN*1 locus (upper panel) and *ADE2* locus (lower panel). All mutations identified at C in *CAN1* or *ADE2* loci of subtelomeric triple reporter (foreground) were analyzed against annotated sequence of *CAN1* or *ADE2* loci, respectively (not against the complete sequence of triple reporter, as in Fig 7B and Fig 7C). Even though the overrepresentation of the motifs was not as significant, as for all the mutations across the triple-reporter sequence, the consensus sequences were very similar. ssDNA, single-strand DNA.
(PPTX)

S8 Fig Signature of aging in human mtDNA. A. pLogo analysis of the aging signature in individual samples of mtDNA from aged brains. The background data set is +/− 20 nucleotides adjacent to the mutation site. The enrichment for the nucleotides above the red arrows is statistically significant, *P* < 0.05. B. Signature of aging in human non-D loop mtDNA. pLogo analysis was performed only for C to T substitutions in non-D loop region of human mtDNA against the background of +/− 20 nucleotides adjacent to the mutation site in heavy strand of the DNA. mtDNA, mitochondrial DNA.
(PPTX)

S1 Table Difference between the frequencies of hydrogen peroxide–Induced and spontaneous mutations measured in strains screened for increased ssDNA mutation sensitivity to redox stress. ^1 1^At least 8 independent spores of a specific genotype were inoculated into rich medium and incubated for 72 hours at room temperature. Cultures were diluted into fresh rich medium and incubated at 37°C for 4 hours. Each culture was split into two, and either mock exposed or exposed to 5 mM hydrogen peroxide for 2 hours. Cells from the cultures were plated on synthetic medium lacking arginine and supplemented with 60 mg/ml of canavanine and, after appropriate dilutions, onto synthetic medium lacking arginine without canavanine. Frequencies of mutations were calculated as the ratio of CanR cells in cultures to the total number of cells. Frequencies of the mutations added by exposure to hydrogen peroxide was calculated by subtraction of the frequency of spontaneous mutations from frequency of induced mutations for each paired measurement for each independent culture in experiment. Median additional frequencies and 95% confidence limits are shown in the table. ^2^Gene functions, relevant to the screen as annotated in *Saccharomyces cerevisiae* Genome Database. ^3^Number of cultures in which absolute frequency of the mutations after exposure to hydrogen peroxide was lower than the absolute frequency of spontaneous mutations. CanR, canavanine-resistant; n/d, not determined; ssDNA, single-strand DNA.
(DOCX)

S2 Table Difference between the frequencies of hydrogen peroxide-induced and spontaneous mutations in dsDNA (midchromosome reporter). ^**1** 1^At least eight independent spores of a specific genotype were inoculated into rich medium and incubated for 72 hours at room temperature. Cultures were diluted into fresh rich medium and incubated at 37°C for 6 hours. Each culture was split in two and either exposed or mock exposed to 5 mM hydrogen peroxide for 2 hours. Cells from the cultures were plated on synthetic medium lacking arginine and supplemented with 60 mg/ml of canavanine and, after appropriate dilutions, onto synthetic medium lacking arginine, without canavanine. Frequencies of mutations were calculated as the ratio of CanR cells in cultures to the total number of cells. Frequencies of the mutations added by exposure to hydrogen peroxide was calculated by subtraction of the frequency of spontaneous mutations from frequency of induced mutations for each paired measurement for each independent culture in experiment. Median additional frequencies and 95% confidence limits are shown in the table. CanR, canavanine-resistant; dsDNA, double-stranded DNA.
(DOCX)

S3 Table Density of mutations identified by sequencing of the triple reporter of CanR Red isolates. ^1^Triple-reporter system allows direct selection for two closely spaced mutations in *CAN1* and *ADE2* loci. Every additional mutation identified by sequencing of the entire reporter sequence was counted as “nonselected.” If three mutations were identified in the reporter sequence of CanR Red isolate, one of them was counted as nonselected; if four mutations were identified in another isolate, two of the mutations were considered nonselected, etc. For instance, in *wt* strain the number of additional mutations was calculated as 15 × 1 + 7 × 2 + 1 × 4 = 32. ^2^Density of nonselected, additional mutations per kb was calculated as a sum of all additional mutations divided to total number of sequenced mutants and divided to the length of the reporter sequence in kb. For instance, for *wt* strain the density of mutation was calculated as 32 ÷ 50 ÷ 9.3 = 0.069 additional mutations per 1 kb. CanR Red, canavanine-resistant red; wt, wild-type.
(DOCX)

S4 Table Fraction of non-GCR events among all the mutational events leading to appearance of CanR Red mutants. A. Number of Ura^+^ colonies of total number CanR Red isolates (in parenthesis). Randomly picked CanR Red isolates, one from a culture of each of genotype, were streaked for single colonies and replica plated onto synthetic medium lacking uracil. Total number of replica plated CanR Red isolates are shown in parenthesis. Consistent with published data following exposure to hydrogen peroxide in all backgrounds, the majority of CanR Red mutants were Ura+, which indicates that such isolates contained multiple mutations in the reporter sequence and were not caused by GCRs because GCR events would have led to a loss of the *URA3* locus as well as to loss of *CAN1* and *ADE2* genes in subtelomeric reporter. There was no significant difference in the fraction of URA+ isolates among different backgrounds (chi-squared test *P* = 0.11). CanR Red, canavanine-resistant red; GCR, gross chromosomal rearrangement.
(DOCX)

S5 Table Catalog of all the sequenced mutations. (XLSX)

S6 Table Estimated frequencies of hydrogen peroxide–induced, closely spaced mutations at a specific nucleotide in CanR Red isolates in ssDNA. ^1^For each genetic background, mutation frequencies at each nucleotide were calculated by multiplication of the median frequency of hydrogen peroxide–induced CanR Red mutations (S2B Fig) to the fraction of the mutations occurring at a specific nucleotide (Fig 4A). CanR Red, canavanine-resistant red; ssDNA, single-strand DNA.
(DOCX)

S7 Table Catalog of the data relevant to analyses of redox stress–induced mutational signatures in human cancers. (XLSX)

S8 Table Measurement of the redox signature mutations in clusters in cancer genomes. (XLSX)

S9 Table Analysis of correlation for enrichment of the redox stress and replicative polymerase mutation signatures. (XLSX)

S10 Table Analysis of the replicative bias of ggCg to ggTg signatures. (XLSX)

S11 Table Primers used in this study. (DOCX)

S1 Data Data underlying Figs 1–6, Fig 9, S1–S3 Figs, S5 Fig and S6 Fig. (XLSX)

## Abbreviations

APOBEC: apolipoprotein B mRNA editing enzyme, catalytic polypeptide-like
CanR Red: canavanine-resistant red
CRC: colorectal carcinoma
dNTP: deoxynucleotide triphosphate
dsDNA: double-stranded DNA
GCR: gross chromosomal rearrangement
LUAD: lung adenocarcinoma
MMS: methyl methanesulfonate
mtDNA: mitochondrial DNA
NAM: nicotinamide
ROS: reactive oxygen species
ssDNA: single-strand DNA
TLS: translesion synthesis
UCEC: uterine corpus endometrial carcinoma
UV: ultraviolet
