# Supplementary figures and images for: Mutational signatures of redox stress in yeast single-strand DNA and of aging in human mitochondrial DNA share a common feature
Source: PLoS Biol. 2019 May 8;17(5):e3000263. doi: 10.1371/journal.pbio.3000263 (PMC6527239; doi:10.1371/journal.pbio.3000263)

## Slide 1
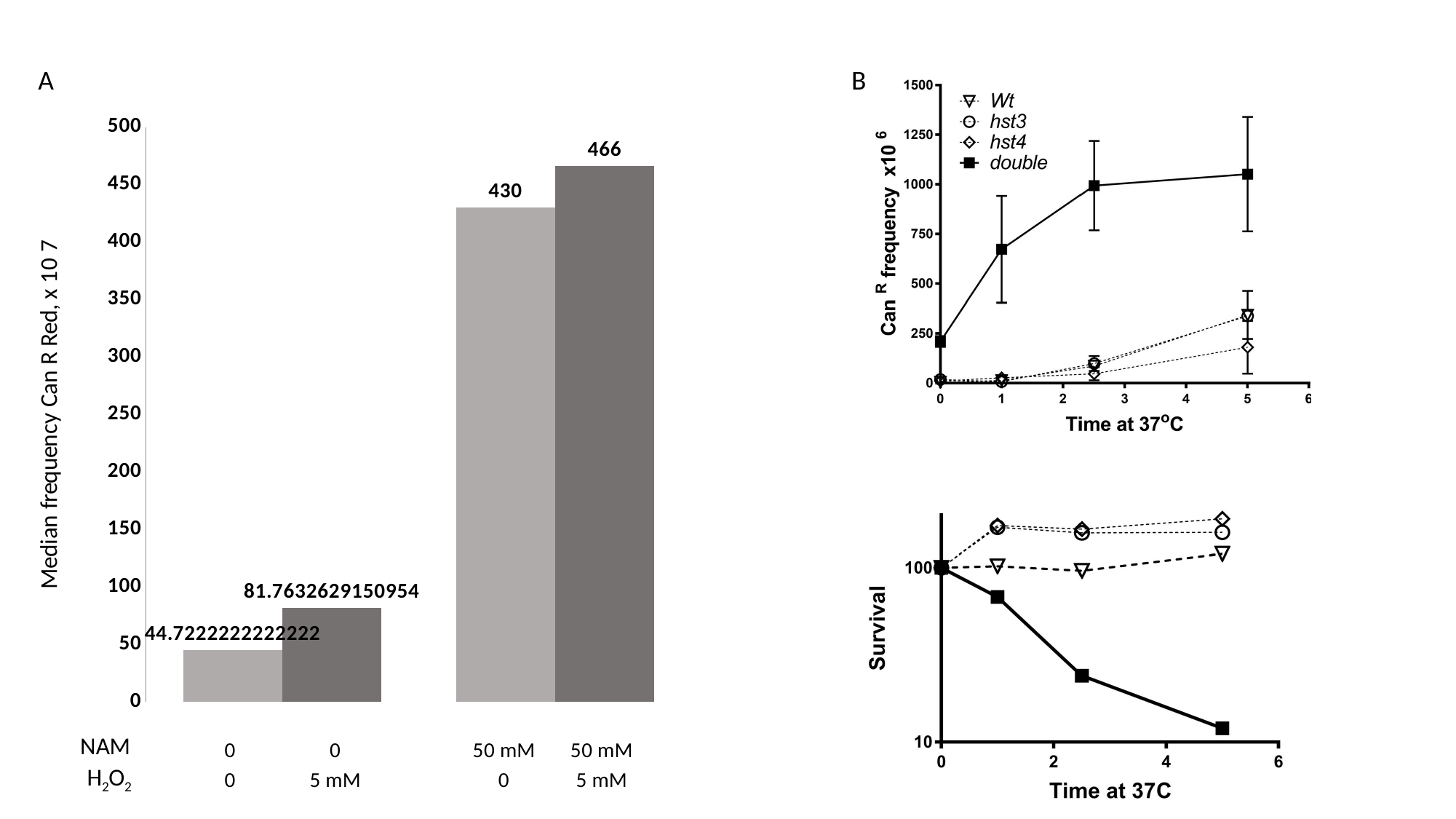

A
B
### Chart
| Category | no peroxide | 5 mM |
|---|---|---|
| No NAM | 44.72222222222222 | 81.7632629150954 |
| 50 mM NAM | 430.0 | 466.0 |

Supplement: S1 Fig — A. Exposure to 50 mM NAM increases frequency of spontaneous but not hydrogen peroxide–induced mutations. All experiments were carried out as described in Materials and methods. Cultures inoculated from independent colonies were incubated with or without NAM in rich medium for 72 hours at room temperature. Cultures were diluted into fresh rich medium and incubated at 37°C for 6 hours. Each culture was split in two and either exposed or mock exposed to 5 mM hydrogen peroxide for 2 hours. Cells from the cultures were plated on synthetic medium with decreased amount of adenine lacking arginine and supplemented with 60 mg/l of canavanine and, after appropriate dilutions, onto synthetic medium lacking arginine without canavanine. Frequency of mutations were calculated as the ratio of CanR Red cells to the total number of cells in cultures. Mutation frequencies for six to 12 independent cultures were measured in each experiment. B. Hst3 or Hst4 histone deacetylases are redundant in protection of ssDNA from oxidative damage; double mutant exhibits increased mutation frequencies and cell killing. Independent spores of each genotype were inoculated into rich medium and incubated at room temperature for 72 hours. Cultures were diluted into fresh rich medium and incubated at 37°C. Aliquots of cultures were taken after before the beginning of incubation at 37°C and after 1, 2.5, and 5 hours of incubation; cells were plated onto complete synthetic medium lacking arginine and supplemented with 60 mg/l of canavanine and, after appropriate dilution, plated onto complete synthetic medium. Mean frequency of CanR and standard errors are shown. See also S1 Data. CanR Red, canavanine-resistant red; H2O2, hydrogen peroxide; NAM, nicotinamide; ssDNA, single-strand DNA. (PPTX) [file pbio.3000263.s001.pptx]

## Slide 1
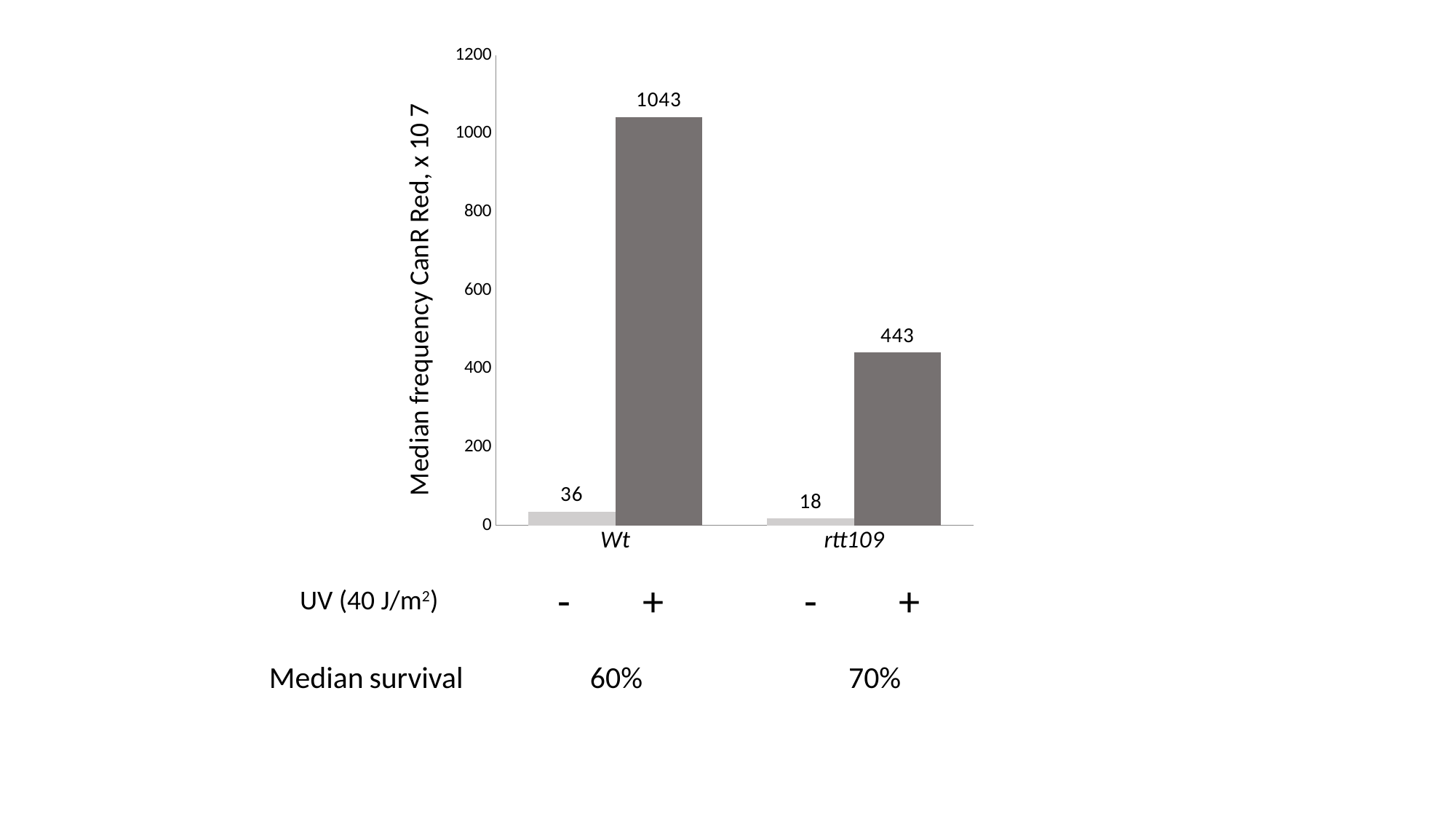

### Chart
| Category | no UV | 40 J/M2 |
|---|---|---|
| Wt | 35.95556054314892 | 1042.9687500000002 |
| rtt109 | 18.037544542816207 | 442.5179211469535 |-
+
-
+
UV (40 J/m2)
Median survival
60%
70%

Supplement: S5 Fig — Eight freshly dissected spores of each genotype were inoculated into rich medium and incubated at 23°C for 72 hours. Cultures were diluted into fresh rich medium and incubated at 37°C for 6 hours. Each culture was split in two and either exposed or mock exposed to UV at 40 J/m2 in Stratalinker 2400 UV Crosslinker (Stratagene). Cells were plated on synthetic medium with decreased amount of adenine, lacking arginine and supplemented with 60 mg/l of canavanine and, after appropriate dilutions, onto synthetic medium lacking arginine, without canavanine. Frequencies of mutations were calculated as the ratio of CanR Red cells to the total number of cells in cultures. Frequencies of mutations in wt strain were significantly higher than in rtt109 strain (P = 0.01, Mann–Whitney test). See also S1 Data. CanR Red, canavanine-resistant red; UV, ultraviolet irradiation; wt, wild-type. (PPTX) [file pbio.3000263.s005.pptx]
