# Supplementary material for: Mutational signatures of redox stress in yeast single-strand DNA and of aging in human mitochondrial DNA share a common feature
Source: PLoS Biol. 2019 May 8;17(5):e3000263. doi: 10.1371/journal.pbio.3000263 (PMC6527239; doi:10.1371/journal.pbio.3000263)
Supplement: S2 Fig — A. Exposure to hydrogen peroxide increases mutation frequencies in dsDNA. Eight to 16 independent, freshly dissected spores of each genotype harboring midchromosome triple reporter were inoculated into rich medium and incubated at 23°C for 72 hours. Cultures were diluted into fresh rich medium and incubated at 37°C for 6 hours. Each culture was split in two and either exposed or mock exposed to 10 mM hydrogen peroxide for 2 hours. Cells from the cultures were plated on synthetic medium lacking arginine and supplemented with 60 mg/l of canavanine and, after appropriate dilutions, onto synthetic medium lacking arginine without canavanine. Frequencies of mutations were calculated as the ratio of CanR cells in cultures to the total number of cells. B. Exposure to hydrogen peroxide increases frequencies of the clustered mutation in ssDNA. Eight to 16 independent, freshly dissected spores of each genotype harboring subtelomeric triple reporter were inoculated into rich medium and incubated at 23°C for 72 hours. Cultures were diluted into fresh rich medium and incubated at 37°C for 6 hours. Each culture was split into two and either exposed or mock exposed to 5 mM hydrogen peroxide for 2 hours. Cells from the cultures were plated on synthetic medium with decreased amount of adenine, lacking arginine and supplemented with 60 mg/ml of canavanine and, after appropriate dilutions, onto synthetic medium lacking arginine, without canavanine. Frequencies of mutations were calculated as the ratio of CanR Red cells to the total number of cells in culture. Whiskers represent 95% confidence interval for median frequency. See also S1 Data. CanR Red, canavanine-resistant red; dsDNA, double-stranded DNA; ssDNA, single-strand DNA. (PPTX) [file pbio.3000263.s002.pptx]

## Slide 1
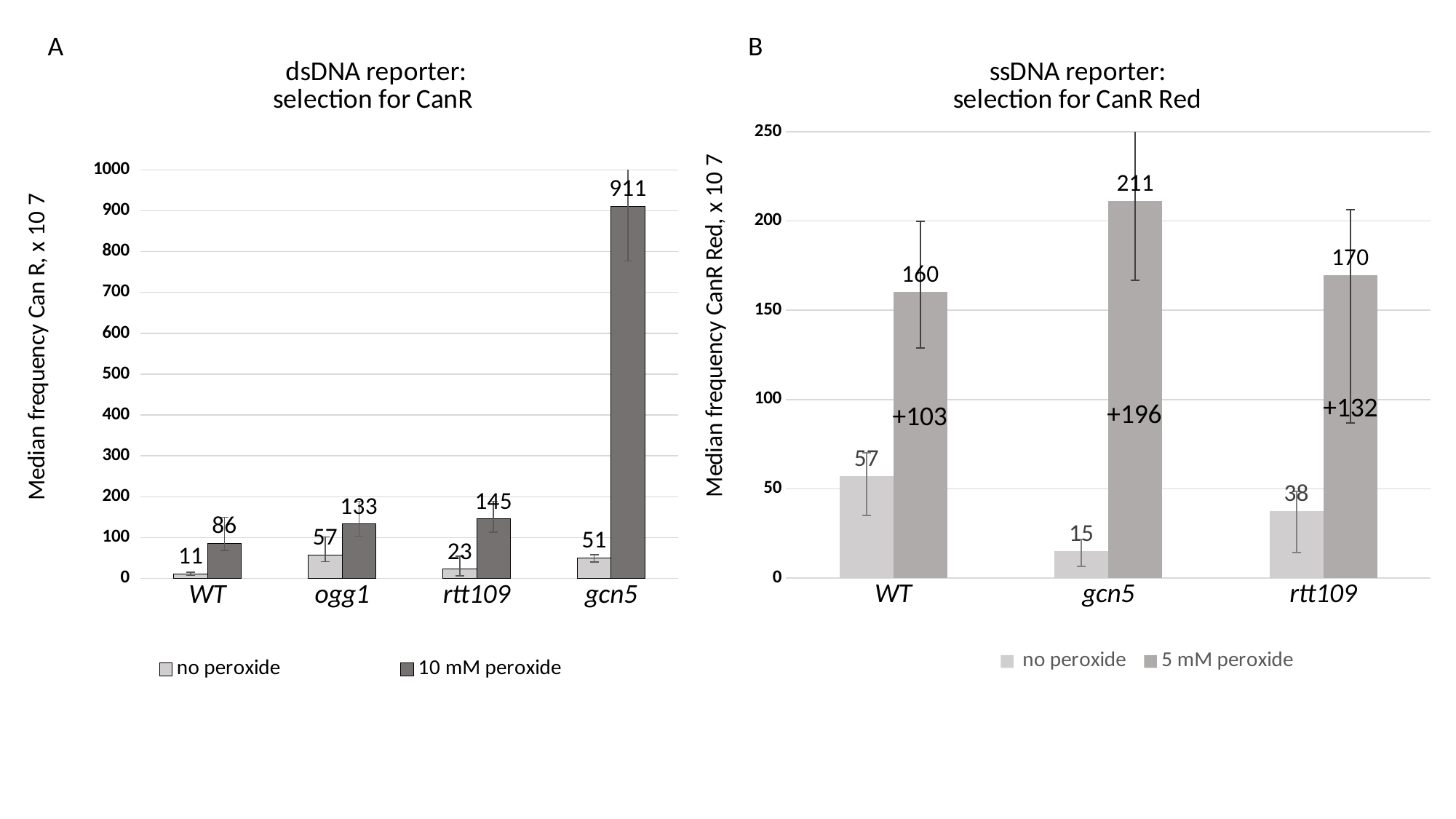

A
B
### Chart
| Category | no peroxide | 10 mM peroxide |
|---|---|---|
| WT | 11.022245762711865 | 85.63536383352972 |
| ogg1 | 57.384987893462466 | 132.96703296703296 |
| rtt109 | 22.792377631087312 | 145.47517921751052 |
| gcn5 | 50.83333333333333 | 911.3906610357326 |
### Chart
| Category | no peroxide | 5 mM peroxide |
|---|---|---|
| WT | 56.88073394495413 | 160.1138587439957 |
| gcn5 | 15.023474178403756 | 211.33088278195928 |
| rtt109 | 37.51460849240358 | 169.67830807876186 |
