# Supplementary material for: Mutational signatures of redox stress in yeast single-strand DNA and of aging in human mitochondrial DNA share a common feature
Source: PLoS Biol. 2019 May 8;17(5):e3000263. doi: 10.1371/journal.pbio.3000263 (PMC6527239; doi:10.1371/journal.pbio.3000263)
Supplement: S3 Fig — A. Fraction of nonselected mutations in CanR Red isolates in rtt109 and gcn5 strains is larger than in wt and ogg1 strains. Schematic presentation of the proportion of mutants with 2, 3, 4, 5, and 7 mutations identified by Sanger sequencing of the subtelomeric reporter sequence (Fig 1A) for corresponding number (n) of CanR Red mutants of each genotype. Fraction of mutants with nonselected mutations (3 and more) for rtt109 and gcn5 strains is significantly higher than that for wt and ogg1 strains (P = 0.04 for one-sided Fisher exact test). B. Number of adjacent mutations with the certain distance between them as a fraction of total number of adjacent mutations. To compare the distribution of closely spaced mutations in rtt109 and gcn5 histone acetylase mutants with the distribution in the strains with nonperturbed chromatin status (wt and ogg1), the data for corresponding groups were combined and presented as percent of mutations with distance between them within the bins of 1 to 400 bp, 401 to 800 bp, 801 to 1,200 bp, and 1,201 to 1,600 bp. Distribution of distances between the groups was significantly different (chi-squared P = 0.04). C. Distribution of the distances between hydrogen peroxide-induced, adjacent mutations in CAN1 and ADE2 loci in triple subtelomeric reporter of CanR Red isolates. Each sphere represents the distance between two adjacent mutations identified by Sanger sequencing of independent CanR Red isolates in wild-type and mutant strains. See also S1 Data. CanR Red, canavanine-resistant red; wt, wild-type. (PPTX) [file pbio.3000263.s003.pptx]

## Slide 1
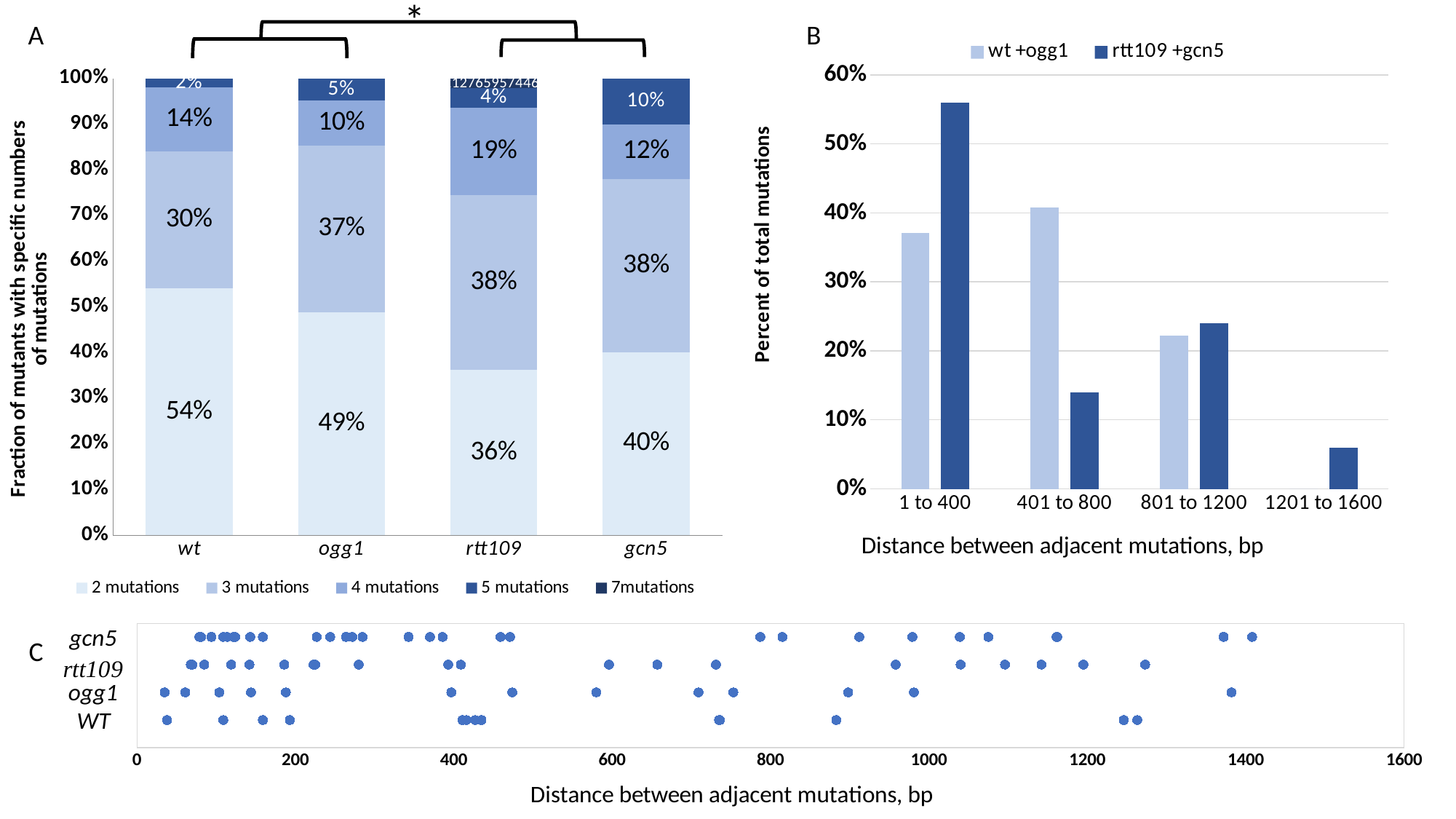

*
### Chart
| Category | 2 mutations | 3 mutations | 4 mutations | 5 mutations | 7mutations |
|---|---|---|---|---|---|
| wt | 0.54 | 0.3 | 0.14 | 0.02 | None |
| ogg1 | 0.4878048780487805 | 0.36585365853658536 | 0.0975609756097561 | 0.04878048780487805 | None |
| rtt109 | 0.3617021276595745 | 0.3829787234042553 | 0.19148936170212766 | 0.0425531914893617 | 0.02127659574468085 |
| gcn5 | 0.4 | 0.38 | 0.12 | 0.1 | None |A
B
### Chart
| Category | wt +ogg1 | rtt109 +gcn5 |
|---|---|---|
| 1 to 400 | 0.37037037037037035 | 0.5599999999999999 |
| 401 to 800 | 0.4074074074074074 | 0.14 |
| 801 to 1200 | 0.2222222222222222 | 0.24 |
| 1201 to 1600 | 0.0 | 0.06 |
### Chart
| Category | |
|---|---|C
