# Supplementary material for: Mutational signatures of redox stress in yeast single-strand DNA and of aging in human mitochondrial DNA share a common feature
Source: PLoS Biol. 2019 May 8;17(5):e3000263. doi: 10.1371/journal.pbio.3000263 (PMC6527239; doi:10.1371/journal.pbio.3000263)
Supplement: S4 Fig — The map of the mutations in CanR Red isolates in wt (A), rtt109 (B), and gcn5 (C) strains. Reference sequence is presented schematically to the right of the map of mutations in wt strains. Red arrows highlight pairs of mutations that are less than 147 bp apart. See also S5 Table. CanR Red, canavanine-resistant red; wt, wild-type. (PPTX) [file pbio.3000263.s004.pptx]

## Slide 1
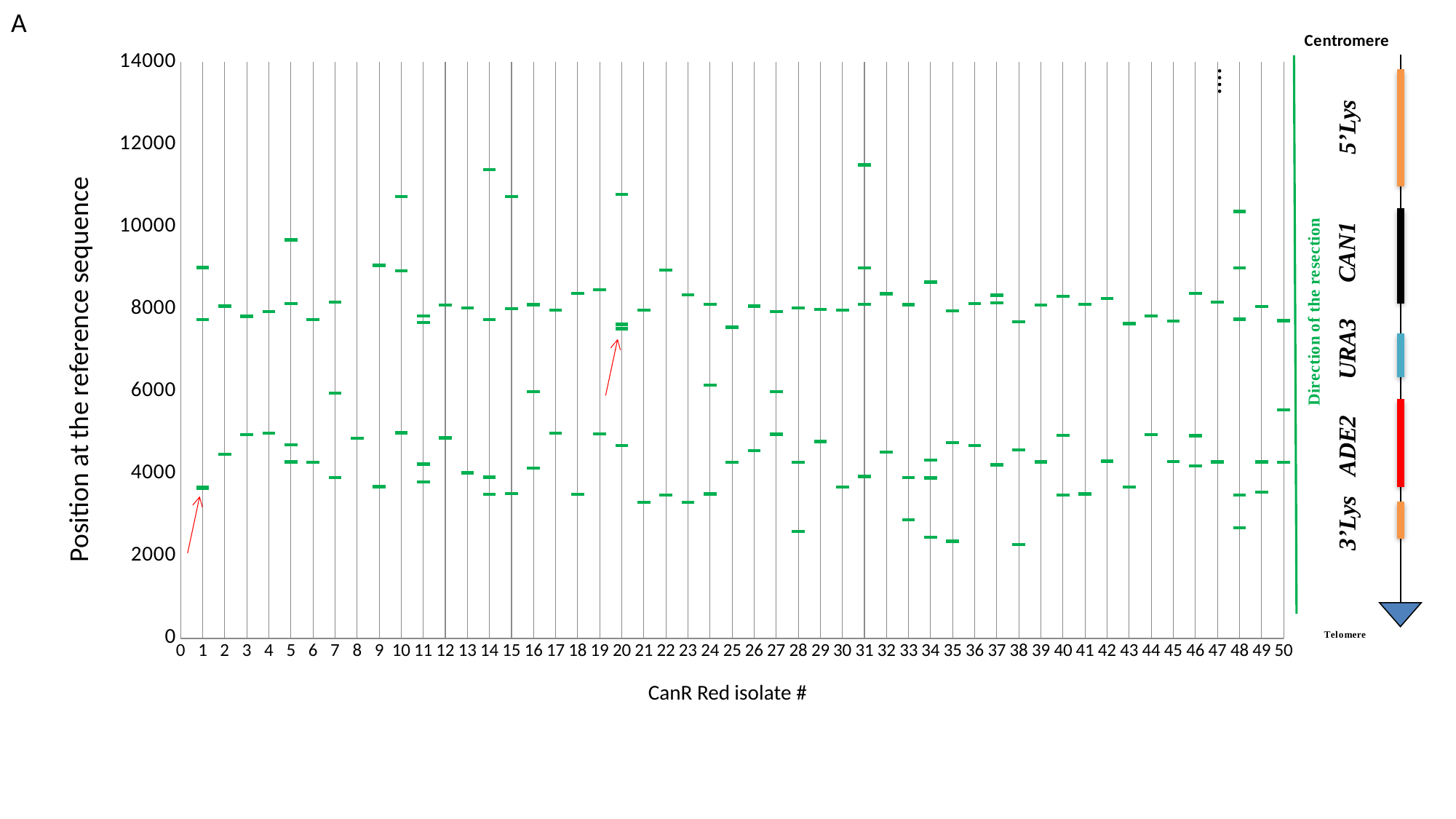

A
### Chart
| Category | |
|---|---|CanR Red isolate #

## Slide 2
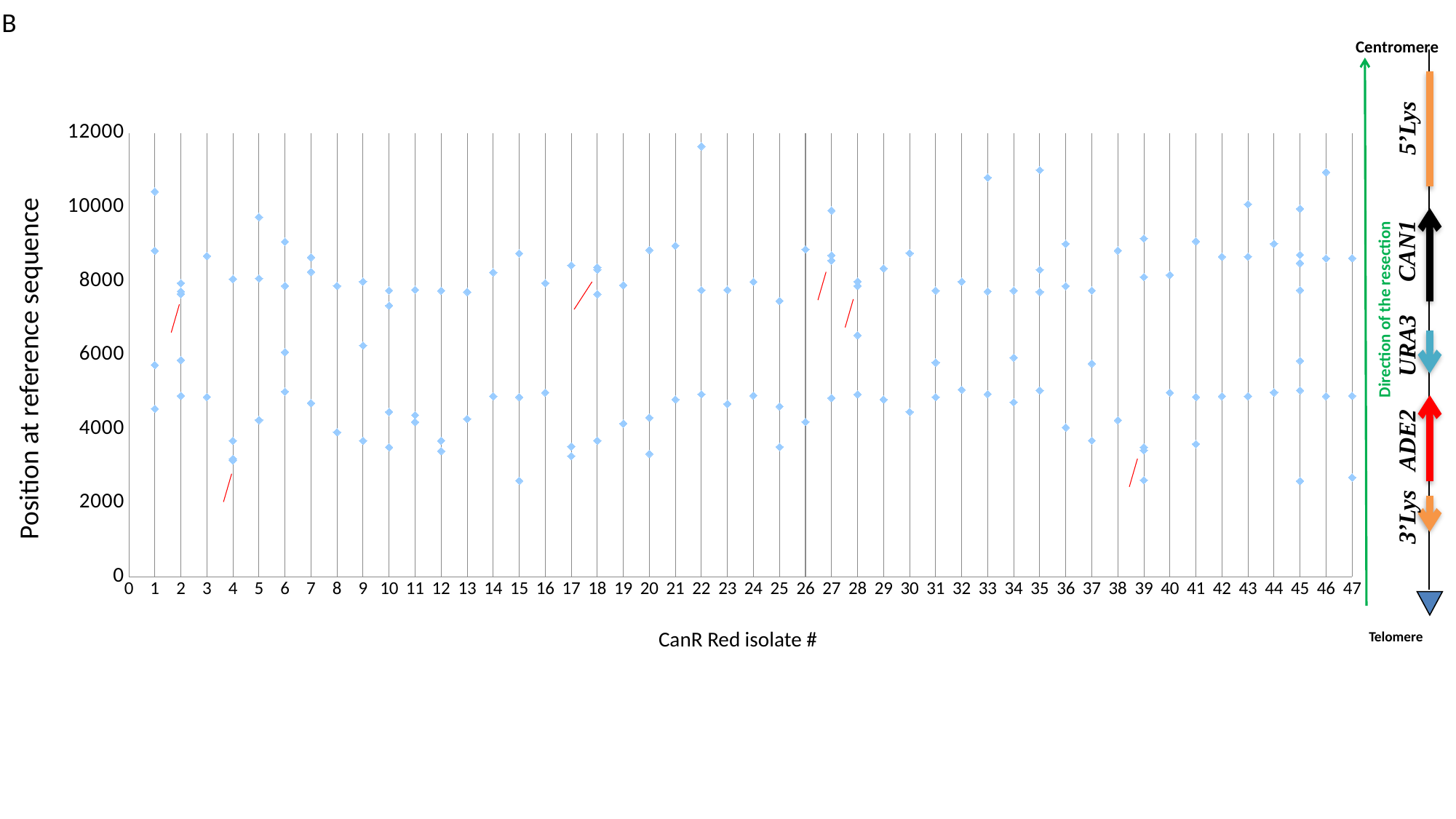

B
Centromere
5’Lys
CAN1
Direction of the resection
URA3
ADE2
3’Lys
Telomere
### Chart
| Category | |
|---|---|Position at reference sequence
CanR Red isolate #

## Slide 3
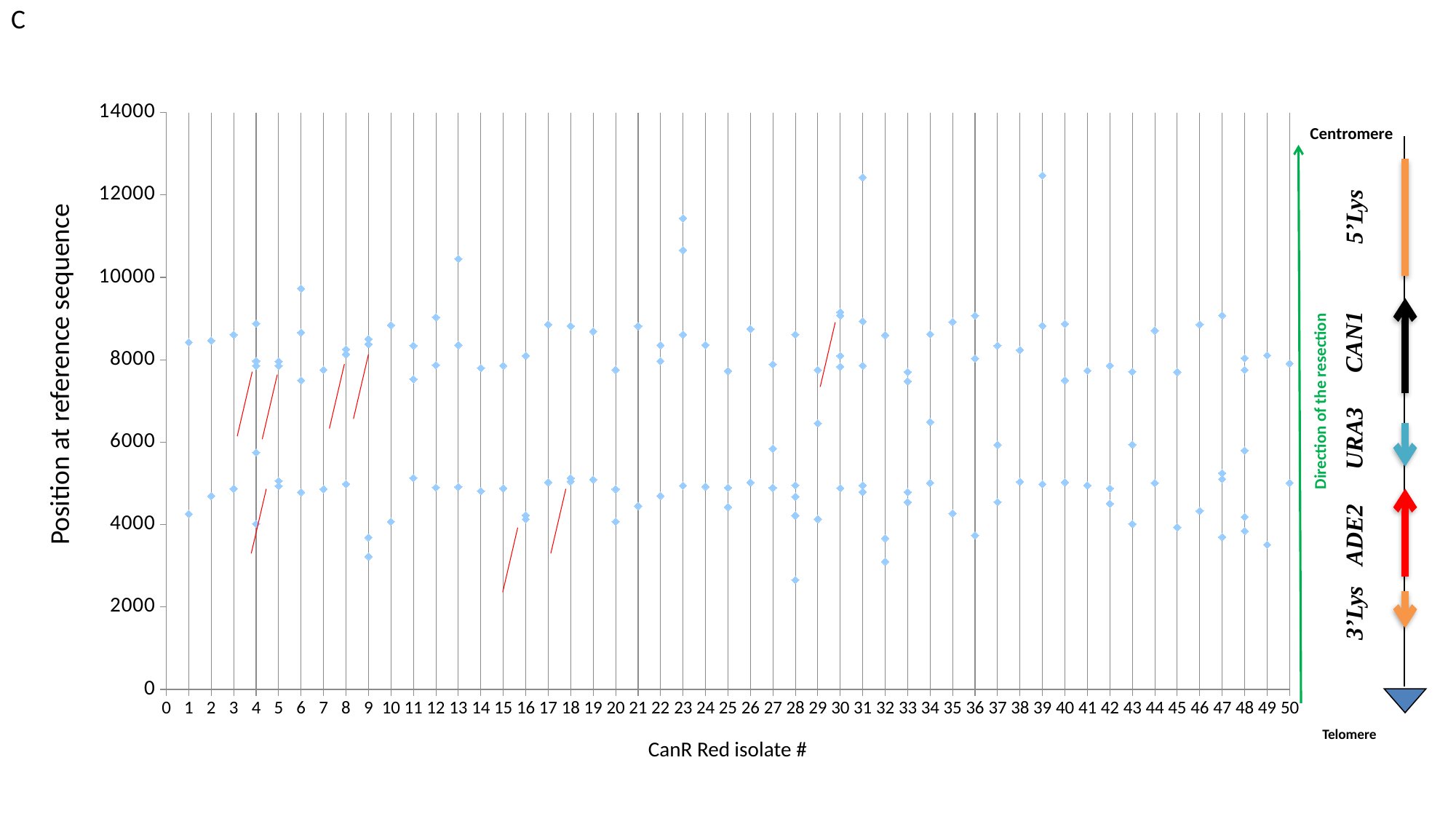

C
### Chart
| Category | |
|---|---|Centromere
5’Lys
CAN1
Direction of the resection
URA3
ADE2
3’Lys
Telomere
Position at reference sequence
CanR Red isolate #
