# Supplementary material for: Mutational signatures of redox stress in yeast single-strand DNA and of aging in human mitochondrial DNA share a common feature
Source: PLoS Biol. 2019 May 8;17(5):e3000263. doi: 10.1371/journal.pbio.3000263 (PMC6527239; doi:10.1371/journal.pbio.3000263)
Supplement: S6 Fig — Exposure to paraquat leads to significant increase in frequencies of CanR single (A) and closely spaced (B) CanR Red mutants in sod1 strains. Four wild-type and 12 sod1 freshly dissected spores of each genotype were inoculated into rich medium and incubated at 23°C for 72 hours. Cultures were diluted into fresh rich medium and incubated at 37°C for 6 hours. Each culture was split into two and either exposed or mock exposed to 50 micromolar paraquat in water for 1.5 hours. Cells from the cultures were plated on synthetic medium with decreased amount of adenine, lacking arginine and supplemented with 60 mg/ml of canavanine, methionine, and lysine (Material and methods) and, after appropriate dilutions, onto synthetic medium lacking arginine without canavanine and supplemented with methionine and lysine. Frequencies of mutations were calculated as the ratio of CanR or CanR Red cells s to the total number of cells in culture. P values were determined by the Mann–Whitney test. See also S1 Data. CanR Red, canavanine-resistant red. (PPTX) [file pbio.3000263.s006.pptx]

## Slide 1
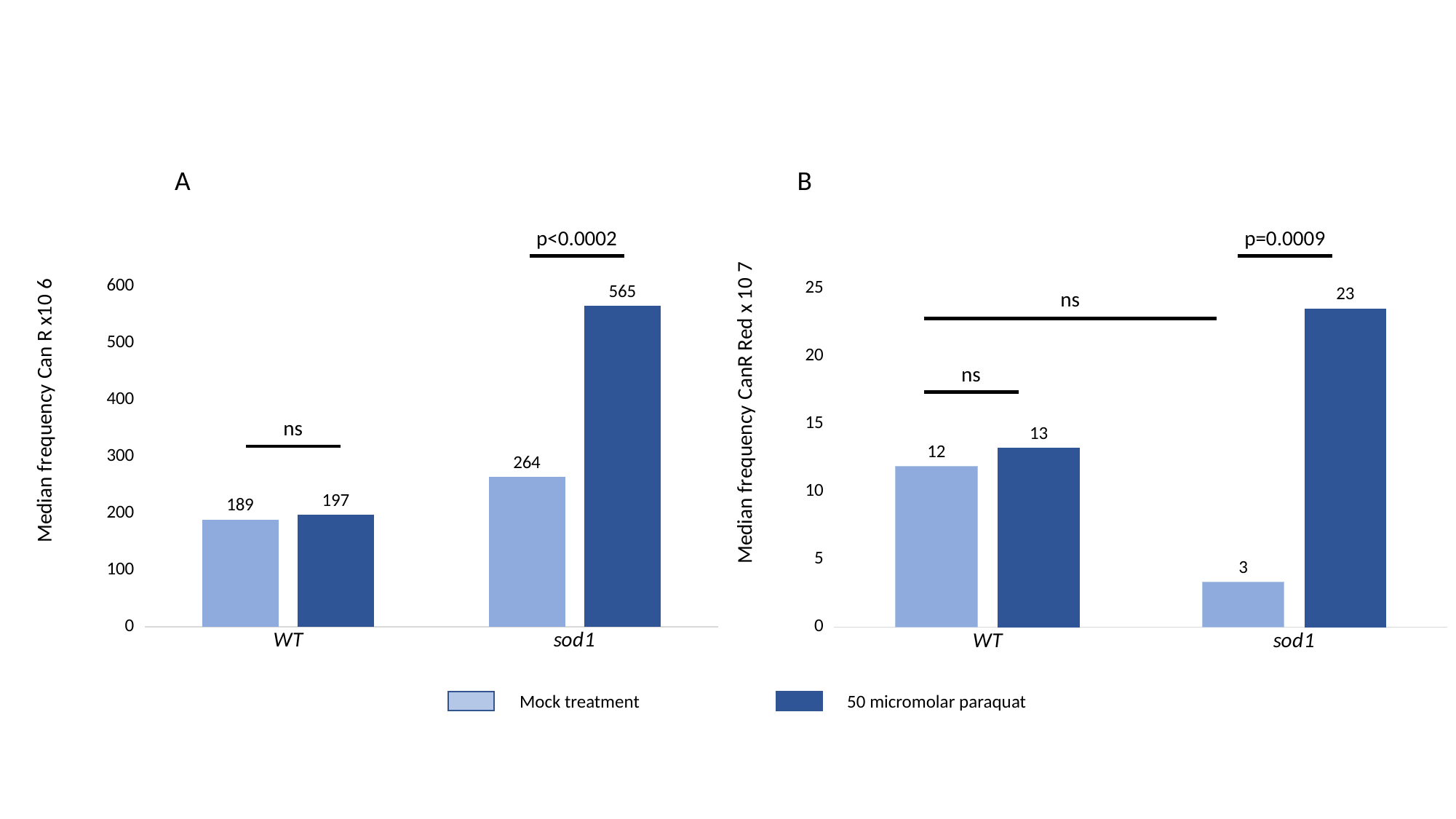

### Chart
| Category | No PQ | 50 uM PQ |
|---|---|---|
| WT | 188.74890071742652 | 197.18462931815242 |
| sod1 | 263.93467023352173 | 565.0093370681604 |A
B
### Chart
| Category | No PQ | 50 uM PQ |
|---|---|---|
| WT | 11.87319255742893 | 13.222926639927545 |
| sod1 | 3.33889816360601 | 23.498964803312628 |p=0.0009
p<0.0002
ns
ns
ns
Mock treatment
50 micromolar paraquat
