# Supplementary material for: Mutational signatures of redox stress in yeast single-strand DNA and of aging in human mitochondrial DNA share a common feature
Source: PLoS Biol. 2019 May 8;17(5):e3000263. doi: 10.1371/journal.pbio.3000263 (PMC6527239; doi:10.1371/journal.pbio.3000263)
Supplement: S7 Fig — pLogo analysis for the motif’s enrichment of a subset of mutations at C in CAN1 locus (upper panel) and ADE2 locus (lower panel). All mutations identified at C in CAN1 or ADE2 loci of subtelomeric triple reporter (foreground) were analyzed against annotated sequence of CAN1 or ADE2 loci, respectively (not against the complete sequence of triple reporter, as in Fig 7B and Fig 7C). Even though the overrepresentation of the motifs was not as significant, as for all the mutations across the triple-reporter sequence, the consensus sequences were very similar. ssDNA, single-strand DNA. (PPTX) [file pbio.3000263.s007.pptx]

## Slide 1
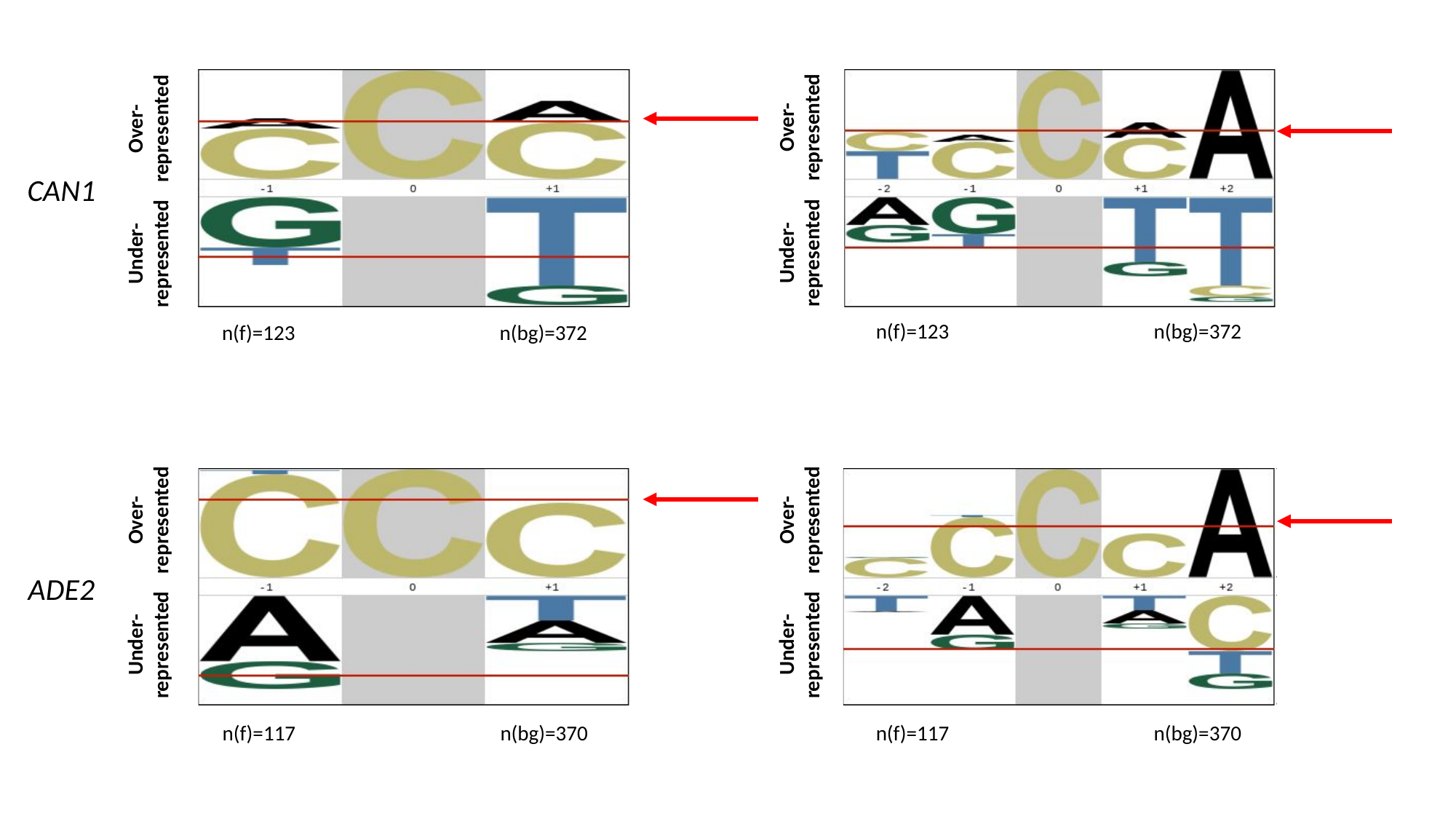

Over-
represented
Under-
represented
Over-
represented
Under-
represented
CAN1
n(f)=123
n(bg)=372
n(f)=123
n(bg)=372
Over-
represented
Under-
represented
Over-
represented
Under-
represented
ADE2
n(f)=117
n(bg)=370
n(f)=117
n(bg)=370
