# Supplementary material for: Mutational signatures of redox stress in yeast single-strand DNA and of aging in human mitochondrial DNA share a common feature
Source: PLoS Biol. 2019 May 8;17(5):e3000263. doi: 10.1371/journal.pbio.3000263 (PMC6527239; doi:10.1371/journal.pbio.3000263)
Supplement: S8 Fig — A. pLogo analysis of the aging signature in individual samples of mtDNA from aged brains. The background data set is +/− 20 nucleotides adjacent to the mutation site. The enrichment for the nucleotides above the red arrows is statistically significant, P < 0.05. B. Signature of aging in human non-D loop mtDNA. pLogo analysis was performed only for C to T substitutions in non-D loop region of human mtDNA against the background of +/− 20 nucleotides adjacent to the mutation site in heavy strand of the DNA. mtDNA, mitochondrial DNA. (PPTX) [file pbio.3000263.s008.pptx]

## Slide 1
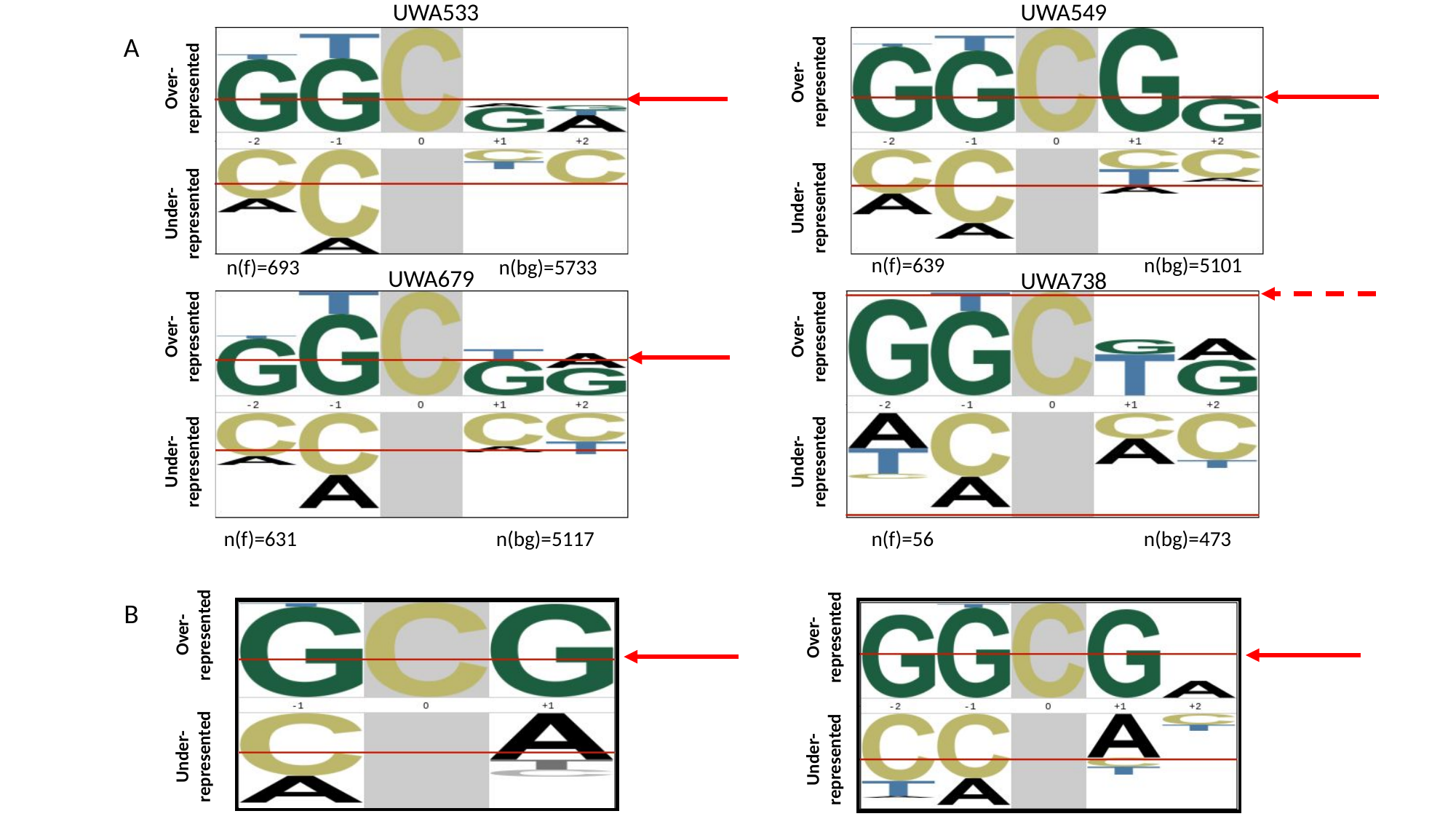

UWA533
UWA549
A
Over-
represented
Under-
represented
Over-
represented
Under-
represented
n(f)=639
n(bg)=5101
n(f)=693
n(bg)=5733
UWA679
UWA738
Over-
represented
Under-
represented
Over-
represented
Under-
represented
n(f)=631
n(bg)=5117
n(f)=56
n(bg)=473
B
Over-
represented
Over-
represented
Under-
represented
Under-
represented
