# Supplementary material for: Mutational signatures of redox stress in yeast single-strand DNA and of aging in human mitochondrial DNA share a common feature
Source: PLoS Biol. 2019 May 8;17(5):e3000263. doi: 10.1371/journal.pbio.3000263 (PMC6527239; doi:10.1371/journal.pbio.3000263)
Supplement: S1 Table — 1 1At least 8 independent spores of a specific genotype were inoculated into rich medium and incubated for 72 hours at room temperature. Cultures were diluted into fresh rich medium and incubated at 37°C for 4 hours. Each culture was split into two, and either mock exposed or exposed to 5 mM hydrogen peroxide for 2 hours. Cells from the cultures were plated on synthetic medium lacking arginine and supplemented with 60 mg/ml of canavanine and, after appropriate dilutions, onto synthetic medium lacking arginine without canavanine. Frequencies of mutations were calculated as the ratio of CanR cells in cultures to the total number of cells. Frequencies of the mutations added by exposure to hydrogen peroxide was calculated by subtraction of the frequency of spontaneous mutations from frequency of induced mutations for each paired measurement for each independent culture in experiment. Median additional frequencies and 95% confidence limits are shown in the table. 2Gene functions, relevant to the screen as annotated in Saccharomyces cerevisiae Genome Database. 3Number of cultures in which absolute frequency of the mutations after exposure to hydrogen peroxide was lower than the absolute frequency of spontaneous mutations. CanR, canavanine-resistant; n/d, not determined; ssDNA, single-strand DNA. (DOCX) [file pbio.3000263.s009.docx]

S1 Table. Difference between the frequencies of hydrogen peroxide-induced and spontaneous mutations measured in strains screened for increased ssDNA mutation sensitivity to redox stress^1)^.

|  | **Absolute difference in frequency of CanR mutants caused by hydrogen peroxide in ssDNA, x 10 ^5^** | | | |  |
| --- | --- | --- | --- | --- | --- |
| **Relevant genotype ^2)^** | **Median** | **95% Confidence limits** | | **Number**  **of independent cultures** | **Number of cultures without** |
|  |  | **Lower** | **Upper** |  | **addition ^3)^** |
| *Wt* | 20 | 16 | 25 | 58 | 0 |
| *cta1* | 23 | 5 | 67 | 8 | 0 |
| *grx2* | 23 | 12 | 54 | 8 | 0 |
| *hsc82* | 23 | 18 | 75 | 8 | 0 |
| *hsp26* | 5 | n/d | 6 | 6 | 1 |
| *mxr1* | 14 | 10 | 17 | 6 | 0 |
| *yap1* | 5 | 2 | 30 | 8 | 0 |
| *cta1 grx2* | 21 | 6 | 40 | 8 | 0 |
| *cta1 hsc82* | 55 | 7 | 296 | 8 | 0 |
| *cta1 sod1* | 29 | 9 | 60 | 14 | 1 |
| *cta1 sod1 hsp26* | 66 | 10 | 81 | 7 | 0 |
| *cta1 sod1 mxr1* | 52 | 36 | 65 | 27 | 0 |
| *cta1 sod1 trx2* | 28 | n/d | 103 | 8 | 1 |
| *rtt109* | 25 | 17 | 39 | 20 | 0 |
| *gcn5* | 36 | 15 | 51 | 27 | 3 |

| *cta1* | Catalase A, breaks down hydrogen peroxide |
| --- | --- |
| *grx2* | Glutaredoxin, thioltransferase, glutathione-dependent disulfide oxidoreductase involved in maintaining redox state of target proteins |
| *hsc82* | Chaperone, heat-inducible |
| *hsp26* | Small heat shock protein with chaperone activity |
| *yap1* | Basic leucine zipper (bZIP) transcription factor; required for oxidative stress tolerance |
| *mxr1* | Methionine-S-sulfoxide reductase; involved in the response to oxidative stress |
| *sod1* | Cytosolic copper-zinc superoxide dismutase; detoxifies superoxide; enters nucleus under oxidative stress |
| *trx2* | Cytoplasmic thioredoxin isoenzyme; part of thioredoxin system which protects cells against oxidative and reductive stress |
