# Supplementary material for: Mutational signatures of redox stress in yeast single-strand DNA and of aging in human mitochondrial DNA share a common feature
Source: PLoS Biol. 2019 May 8;17(5):e3000263. doi: 10.1371/journal.pbio.3000263 (PMC6527239; doi:10.1371/journal.pbio.3000263)
Supplement: S2 Table — 1 1At least eight independent spores of a specific genotype were inoculated into rich medium and incubated for 72 hours at room temperature. Cultures were diluted into fresh rich medium and incubated at 37°C for 6 hours. Each culture was split in two and either exposed or mock exposed to 5 mM hydrogen peroxide for 2 hours. Cells from the cultures were plated on synthetic medium lacking arginine and supplemented with 60 mg/ml of canavanine and, after appropriate dilutions, onto synthetic medium lacking arginine, without canavanine. Frequencies of mutations were calculated as the ratio of CanR cells in cultures to the total number of cells. Frequencies of the mutations added by exposure to hydrogen peroxide was calculated by subtraction of the frequency of spontaneous mutations from frequency of induced mutations for each paired measurement for each independent culture in experiment. Median additional frequencies and 95% confidence limits are shown in the table. CanR, canavanine-resistant; dsDNA, double-stranded DNA. (DOCX) [file pbio.3000263.s010.docx]

S2 Table.

|  | **Absolute increase in CanR mutation frequency caused by hydrogen peroxide in dsDNA, x10 ^6^** | | | |  |
| --- | --- | --- | --- | --- | --- |
| **Relevant genotype** | **Median** | **95% Confidence limits** | | **Number**  **of independent cultures** | **Number of cultures without** |
|  |  | **Lower** | **Upper** |  | **addition** |
| *Wt* | 7.7 | 5.8 | 13.8 | 16 | 0 |
| *ogg1* | 7.0 | 4.9 | 10.9 | 7 | 0 |
| *rtt109* | 12.3 | 5.9 | 47.5 | 8 | 0 |
| *gcn5* | 86.1 | 71.4 | 98.1 | 15 | 0 |
