# Supplementary material for: Mutational signatures of redox stress in yeast single-strand DNA and of aging in human mitochondrial DNA share a common feature
Source: PLoS Biol. 2019 May 8;17(5):e3000263. doi: 10.1371/journal.pbio.3000263 (PMC6527239; doi:10.1371/journal.pbio.3000263)
Supplement: S3 Table — 1Triple-reporter system allows direct selection for two closely spaced mutations in CAN1 and ADE2 loci. Every additional mutation identified by sequencing of the entire reporter sequence was counted as “nonselected.” If three mutations were identified in the reporter sequence of CanR Red isolate, one of them was counted as nonselected; if four mutations were identified in another isolate, two of the mutations were considered nonselected, etc. For instance, in wt strain the number of additional mutations was calculated as 15 × 1 + 7 × 2 + 1 × 4 = 32. 2Density of nonselected, additional mutations per kb was calculated as a sum of all additional mutations divided to total number of sequenced mutants and divided to the length of the reporter sequence in kb. For instance, for wt strain the density of mutation was calculated as 32 ÷ 50 ÷ 9.3 = 0.069 additional mutations per 1 kb. CanR Red, canavanine-resistant red; wt, wild-type. (DOCX) [file pbio.3000263.s011.docx]

S3 Table.

|  | **Relevant genotype** | | |  |  | |
| --- | --- | --- | --- | --- | --- | --- |
|  | ***wt*** | ***ogg1*** | ***rtt109*** | | | ***gcn5*** |
| Number of mutations identified in triple reporter |  |  |  | | |  |
| 2 mutations | 27 | 20 | 17 | | | 20 |
| 3 mutations | 15 | 15 | 18 | | | 19 |
| 4 mutations | 7 | 4 | 9 | | | 6 |
| 5 mutations | 1 | 2 | 2 | | | 5 |
| 7 mutations | 0 | 0 | 1 | | | 0 |
| Total number of non-selected mutations ^1)^ | 32 | 29 | 47 | | | 46 |
| Total number of mutations | 132 | 111 | 141 | | | 146 |
| # Mutants sequenced | 50 | 41 | 47 | | | 50 |
| Reporter size, kb | 9.3 | 9.3 | 9.3 | | | 9.3 |
| Density, mutation per kb ^2)^ | 0.069 | 0.062 | 0.101 | | | 0.099 |
| One additional mutation per kb | 14.5 | 16.1 | 9.3 | | | 10.1 |
