# Supplementary material for: Mutational signatures of redox stress in yeast single-strand DNA and of aging in human mitochondrial DNA share a common feature
Source: PLoS Biol. 2019 May 8;17(5):e3000263. doi: 10.1371/journal.pbio.3000263 (PMC6527239; doi:10.1371/journal.pbio.3000263)
Supplement: S4 Table — A. Number of Ura+ colonies of total number CanR Red isolates (in parenthesis). Randomly picked CanR Red isolates, one from a culture of each of genotype, were streaked for single colonies and replica plated onto synthetic medium lacking uracil. Total number of replica plated CanR Red isolates are shown in parenthesis. Consistent with published data following exposure to hydrogen peroxide in all backgrounds, the majority of CanR Red mutants were Ura+, which indicates that such isolates contained multiple mutations in the reporter sequence and were not caused by GCRs because GCR events would have led to a loss of the URA3 locus as well as to loss of CAN1 and ADE2 genes in subtelomeric reporter. There was no significant difference in the fraction of URA+ isolates among different backgrounds (chi-squared test P = 0.11). CanR Red, canavanine-resistant red; GCR, gross chromosomal rearrangement. (DOCX) [file pbio.3000263.s012.docx]

S4 Table.

|  | Number of Ura^+^ colonies among CanR Red ^a)^ | |
| --- | --- | --- |
|  | No hydrogen peroxide | 5mM hydrogen peroxide |
| *Wt* | 4 (31) | 36 (65) |
| *pol2-4* | 4 (27) | 24 (33) |
| *pol3-5DV* | 8 (9) | 11 (14) |
