# Supplementary material for: Mutational signatures of redox stress in yeast single-strand DNA and of aging in human mitochondrial DNA share a common feature
Source: PLoS Biol. 2019 May 8;17(5):e3000263. doi: 10.1371/journal.pbio.3000263 (PMC6527239; doi:10.1371/journal.pbio.3000263)
Supplement: S6 Table — 1For each genetic background, mutation frequencies at each nucleotide were calculated by multiplication of the median frequency of hydrogen peroxide–induced CanR Red mutations (S2B Fig) to the fraction of the mutations occurring at a specific nucleotide (Fig 4A). CanR Red, canavanine-resistant red; ssDNA, single-strand DNA. (DOCX) [file pbio.3000263.s014.docx]

S6 Table.

|  | Estimated frequency of mutations ^1)^, x10 ^7^ | | | | |
| --- | --- | --- | --- | --- | --- |
|  | G | C | A | T | other |
| *wt* | 23 | 88 | 28 | 10 | 11 |
| *ogg1* | 38 | 137 | 38 | 29 | 24 |
| *gcn5* | 31 | 106 | 42 | 15 | 18 |
| *rtt109* | 30 | 88 | 20 | 21 | 11 |

^1)^ For each genetic background mutation frequencies at each nucleotide and were calculated by multiplication of the median frequency of hydrogen peroxide-induced CanR Red mutations (S2B Fig) to the fraction of the mutations occurring at a specific nucleotide (Fig 4A)
