# Supplementary material for: Mutational signatures of redox stress in yeast single-strand DNA and of aging in human mitochondrial DNA share a common feature
Source: PLoS Biol. 2019 May 8;17(5):e3000263. doi: 10.1371/journal.pbio.3000263 (PMC6527239; doi:10.1371/journal.pbio.3000263)
Supplement: S11 Table — (DOCX) [file pbio.3000263.s019.docx]

S11 Table.

| **Primer Name** | **Sequence** | **Amplification region/Locus sequenced** |
| --- | --- | --- |
| Primers used to amplify subtelomeric reporter sequence | | |
| oDG_89 | TTAAGCTGCTGCGGAGCT | *3' LYS2* |
| oDG_90' | AGCCATGCAACAAGAGTC | *3' LYS2* |
| oDG_107 | ACGGCGGCTAAGCTCATAAC | *3' LYS2_ADE2* junction |
| oDG_108 | GCTGCCTCAACAATCATACG | *3' LYS2_ADE2* junction |
| oKC073 | CGAACCGGGTAATACTAAGTG | *ADE2* |
| oKC074 | GCCAAATTGAGGGATCTTATG | *ADE2* |
| oDG_77 | TCTTGCAAATGCAGCTTCTTC | *URA3* |
| oDG_78 | AAGAACGAAGGAAGGAGCACA | *URA3* |
| oKC062 | AGGGTGAGAATGCGAAATG | *CAN1* |
| oKC142 | GGTTGCGAACAGAGTAAACC | *CAN1* |
| oDG_91 | CGTGGGCAAACACTTTGAAA | *5' LYS2* |
| oDG_92 | TGACTAACGAAAAGGTCTGGA | *5' LYS2* |
| Primers used for sequencing of subtelomeric triple reporter loci | | |
| oDG_91 | CGTGGGCAAACACTTTGAAA | *5' LYS* |
| oDG_92 | TGACTAACGAAAAGGTCTGGA | *5' LYS* |
| seqDG_101 | CGTGGGCAAACACTTTGA | *5' LYS* |
| seqDG_102 | ACTTGAAAATTGTGCCCA | *5' LYS* |
| seqDG_103 | CCATAACCACAATCAAGG | *5' LYS* |
| seqDG_106 | TGCAACCATACTTACTCA | *5' LYS* |
| seqDG_108 | CCACACCCCTAGAAGAAT | *5' LYS* |
| seqDG_111 | TGACTAACGAAAAGGTCT | *5' LYS* |
| seqDG_112 | AACAATGAGTTGAACAAG | *5' LYS* |
| seqDG_113 | CGCTAAGCGATCCATCCA | *5' LYS* |
| seqDG_114 | CTTCTAGGGGTGTGGATT | *5' LYS* |
| seqDG_115 | ACAAAGACACCAGAACAG | *5' LYS* |
| seqDG_116 | GTAAGTATGGTTGCACAG | *5' LYS* |
| seqDG_117 | AGCTACTAGTTGTTAACA | *5' LYS* |
| seqDG_118 | AAGTTAAAATTCGTGGGT | *5' LYS* |
| seqDG_119 | TGATTGTGGTTATGGATA | *5' LYS* |
| seqDG_120 | GCACAATTTTCAAGTATC | *5' LYS* |
| oKC062 | AGGGTGAGAATGCGAAATG | *CAN1* |
| seqDG_83 | CAAATTCAAAAGAAGACG | *CAN1* |
| seqDG_84 | ACGCAGTCCTTGGGTGAA | *CAN1* |
| seqDG_86 | TTGGTCTATCAAAGAACA | *CAN1* |
| seqDG_89 | AACTCGTCACGAGAGATG | *CAN1* |
| seqDG_91 | TTTGACAGGGAACAAGTT | *CAN1* |
| oKC142 | GGTTGCGAACAGAGTAAACC | *CAN1* |
| oKC089 | GGTATTTCACACCGCATAG | *URA3* |
| oKC090 | GGTAATCTCCGAACAGAAG | *URA3* |
| SAR353 | CTTAGCATCCCTTCCCTTTG | *URA3* |
| seqDG_78 | CTCCAGTAGATAGGGAGC | *URA3* |
| SAR354 | GAAGAACGAAGGAAGGAGCACA | *URA3* |
| seqDG_81 | CGGGTGTATACAGAATAG | *URA3* |
| oKC073 | CGAACCGGGTAATACTAAGTG | *ADE2* |
| oKC074 | GCCAAATTGAGGGATCTTATG | *ADE2* |
| seqKC001 | GTTGAGGCAGCAAACAGG | *ADE2* |
| seqKC003 | CCAAGGCCTCACAACTCTG | *ADE2* |
| seqKC006 | GGCACACCGATGACAGGAAG | *ADE2* |
| seqKC007 | GTTCACATTCCGCCATACTG | *ADE2* |
| seqKC008 | AGTGACGCAAGCATCAATGG | *ADE2* |
| SAR337 | AGGACTTTGGCATACGATGG | *ADE2* |
| SAR335 | ACTCTGACTTGCCGGTAATG | *ADE2* |
| oKC109new | GTAAGCTGCTGCGGAGCT | *3'LYS* |
| oKC110 | AGCCATGCAACAAGAGTC | *3'LYS* |
| seqDG_94 | AGGATACAACGCATTTTC | *3'LYS* |
| seqDG_99 | CGAAAATTCCGTGAATAT | *3'LYS* |
| oDG_107 | ACGGCGGCTAAGCTCATAAC | *3'LYS -ADE2 junction* |
| oDG_108 | GCTGCCTCAACAATCATACG | *3'LYS -ADE2 junction* |
